# Coupling metal stable isotope compositions and X-ray absorption spectroscopy to study metal pathways in soil–plant systems: a mini review

**DOI:** 10.1093/mtomcs/mfad016

**Published:** 2023-03-09

**Authors:** Anne Marie Aucour, Géraldine Sarret, Hester Blommaert, Matthias Wiggenhauser

**Affiliations:** Université de Lyon, Université Lyon 1, ENS de Lyon, CNRS, UMR 5276 LGL-TPE, F-69622, Villeurbanne, France; Université Grenoble Alpes, Université Savoie Mont Blanc, CNRS, IRD, Université G. Eiffel, ISTerre, 38058 Grenoble, Cedex 9, France; Université Grenoble Alpes, Université Savoie Mont Blanc, CNRS, IRD, Université G. Eiffel, ISTerre, 38058 Grenoble, Cedex 9, France; Institute of Agricultural Sciences, ETH Zurich, Eschikon 33, CH-8315, Lindau, Switzerland

**Keywords:** X-ray absorption spectroscopy, stable isotopes, speciation, trace metal, micronutrient, contaminant

## Abstract

Excess and limited trace metal contents in soils and plants can limit crop yields and pose a risk for the environment and human health. This mini-review reports on the emerging approach of combining X-ray absorption spectroscopy (XAS) with isotope analyses to improve the understanding of metal speciation and dynamics in soil–plant systems. In soils and their components, shifts in isotope compositions could be in some cases linked to changing metal speciation and thereby provide information on processes that control the phytoavailability of metals. In plants, the XAS-isotope approach has potential to improve the understanding of how complex interactions of metal speciation, redox processes, and membrane transport control metal uptake and translocation to edible plant parts. Yet, the XAS-isotope approach proves to be in a rather exploratory phase, and many research gaps remain. Such limitations can be overcome by methodological improvements and combining the approach with molecular biology and modelling approaches.

## Graphical Abstract

**Graphical Abstract:**
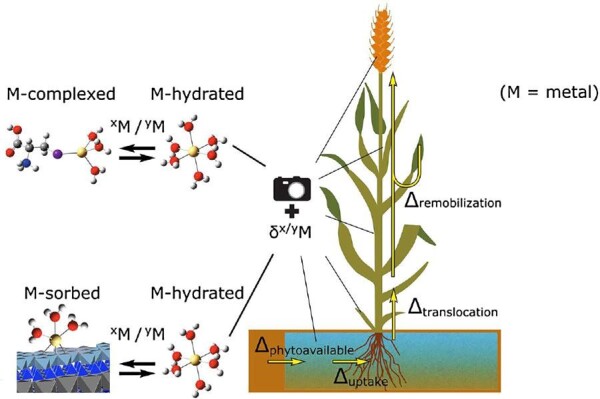
On the pathway of metals (M) from the soil to edible parts of plants, X-ray absorption spectroscopy provides snapshots of metal speciation in the various compartments, while isotope ratios (δ) contain information on biological and physicochemical processes along this pathway.

## Introduction

Understanding the fate of metals in soils and soil–plant systems is crucial to limit environmental and health risks that are associated with trace metals. In many regions, trace metal concentrations in agricultural soils are critically high, and in some regions, the concentrations are further increasing.^1,2^ Worldwide, new legislations on metal contents in fertilizers, biosolids, and crops are implemented to decrease exposure.^3,4^ In parallel, micronutrient deficiency in humans (Fe, Zn, and Se) is a major health issue that affects far more people than metal contamination.^5^ Hence, improving food quality by increasing the content of micronutrients in crops and reducing the exposure of contaminants is a major challenge to sustain food security in the coming decades. This challenge demands knowledge on biogeochemical processes that control the fate of metals in the soil–crop–human continuum and involves different disciplines such as environmental geochemistry, agronomy, and plant physiology.

Metal stable isotopes in natural abundance are increasingly used in environmental sciences to identify sources and to understand biogeochemical processes.^6–9^ Stable isotope fractionation can be divided into kinetic and equilibrium isotope effects. In soil–plant systems, examples of kinetic effects can be the diffusion of metals in the soil solution,^10^ the fast unidirectional transport mediated by membrane proteins,^11^ and enzymatically controlled reactions.^6^ Kinetic processes tend to favour light isotopes, and the extent of the isotope fractionation strongly depends on the progress of the reaction (see section ‘Basics of isotopes’). Equilibrium isotope fractionation is defined for a reaction between two chemical species of a metal when the isotope equilibrium is reached for the reaction. Equilibrium effects are driven by vibrational energy differences in the bonding environments of the two species. Equilibrium isotope fractionation thus depends on the oxidation state, the type of ligand that complexes the metal, and the coordination number.^12,13^ Qualitative rules of thumb predict an enrichment of heavier isotopes in the metal species with a higher oxidation state, a lower coordination number, and a shorter bond length. In this review, we examine to what extent these rules for different isotope fractionation modes are corroborated experimentally and can be applied in soil–plant systems.

The speciation or chemical form of a metal is a key parameter that controls its bioavailability and toxicity. X-ray absorption spectroscopy (XAS) became a widely used tool to study changes in the speciation of elements in environmental samples.^14,15^ Its main strengths include its element selectivity, its ability to identify ordered and disordered phases, and its ability to provide an average speciation on a metal in the sample.^14,15^ In the last decade, XAS and metal stable isotope geochemistry have been combined to investigate compartments that are relevant for soil–plant systems. To study metal associations on single phases such as sorption of metals to minerals, the information provided by XAS on metal speciation, coordination, and bond length can assist the interpretation of the equilibrium isotope fractionation of the metals.^16^ In complex systems such as soils and plants, XAS provides snapshots of metal speciation, while the isotope compositions of a metal can provide information on pathways and the dynamics of the metals in plants.^17,18^

Here, we provide a critical mini review of recent studies that have combined XAS and isotope analyses to investigate sorption and precipitation processes of metals in compartments that are relevant for soil–plant systems. We include transition metals (Zn, Cd, Cu, and Ni) and alkali metals like K. In a first step, we review studies that applied the combined XAS-isotope approach to single phases, which mostly comprise XAS speciation and equilibrium isotope fractionation for metal sorption on single mineral phases or complexation with single organic ligands. In a second step, we review results on soils and plants for which we provide basic knowledge for XAS and isotope fractionation in soil–plant systems, and discuss the links between the processes that drive changes in metal speciation and isotope fractionation. Finally, we point out the current limitations of the complementarity of the two techniques, give methodological recommendations, and discuss future prospects for the coupled XAS-isotope approach.

### Basics of isotopes

The isotope composition of a sample is reported relative to a reference using the δ notation (exemplified for Cd with NIST 3108 as reference):


(1) \begin{eqnarray*}
{{\mathrm{\delta }}}^{114/110}{\mathrm{Cd = }}\left[ {\frac{{{{{(}^{114}{\mathrm{Cd}}{{\mathrm{/}}}^{110}{\mathrm{Cd}})}}_{{\mathrm{sample}}}}}{{{{{(}^{114}{\mathrm{Cd}}{{\mathrm{/}}}^{110}{\mathrm{Cd}})}}_{{\mathrm{NIST}}\,{\mathrm{3108}}}}} - 1} \right] \times 1000.
\end{eqnarray*}

For a compartment composed of several sub-compartments sampled and analysed separately, an isotope mass balance is applied. For example, a plant can be divided into roots, leaves, branches, and fruits. Hence, the isotope composition of the whole plant is calculated by integrating the mass and isotope composition of each compartment into a weighted mean calculation.^19^ Hence, the biomass of the individual plant parts should be measured, or estimated in case the entire biomass cannot be destructively sampled (e.g. for trees).^20^ This isotope composition for the whole plant represents the δ-value of incorporated metals into the plant, assuming that no biomass and metals were lost from the plant. Depending on the definition of ‘whole plant’ and ‘whole root’, root external metal precipitates and sorbed species in the root apoplast may be included or excluded by using chemical extractions (see section ‘The soil–soil solution–plant interfaces’).

The isotope fractionation between two chemical species of a metal is expressed with the isotope fractionation factor α_A–B_ = R_A_/*R*_B_. In this equation, *R*_A_ and *R*_B_ denote the isotope ratios of species A and B. As α is generally close to 1, (α_A–B_- 1) 10^3^ is ≈ δ_A_–δ_B_. The latter can be denoted as Δ_A–B_ in per mil and provides a convenient approximation for the isotope fractionation. For Cd, this approximation is expressed as


(2) \begin{eqnarray*}
{{\mathrm{\Delta }}}^{{\mathrm{114/110}}}{\mathrm{C}}{{\mathrm{d}}}_{{\mathrm{A-B}}}{\mathrm{ = }}{{\mathrm{\delta }}}^{{\mathrm{114/110}}}{\mathrm{C}}{{\mathrm{d}}}_{\mathrm{A}}{\mathrm{-}}{{\mathrm{\delta }}}^{{\mathrm{114/110}}}{\mathrm{C}}{{\mathrm{d}}}_{\mathrm{B}}.
\end{eqnarray*}

The equilibrium isotope fractionation Δ_A–B_ between two chemical species of a metal reported in this review was experimentally determined or estimated using *ab initio* calculations. The isotope fractionation factor between sorbed and aqueous metal (Δ_sorbed–aqueous_) (see section ‘Interactions of metals with minerals and humic acids’) was determined when isotope equilibrium was reached. However, it can be challenging to establish equilibrium isotope fractionation in sorption experiments, and possible kinetic effects and metal speciation in solution may influence the isotope fractionation observed. This point is comprehensively discussed for sorption experiments in Komárek et al. (2021).^16^

In a unidirectional reaction, the product is removed from the reactant pool and induces a kinetic isotope fractionation Δ_kin_. For such a reaction, the isotope difference between the reactant and the instantaneous product is not the same as the isotope difference between the final product and the residual reactant. The isotope composition of the latter strongly depends on the progress of the reaction, and can be modelled with Rayleigh equations (Fig. S1). Once the reaction is completed, the final product has a similar isotope composition as the initial reactant. The complex biological processes that fractionate isotopes in plant-soil systems likely include kinetic and equilibrium effects.

The difference in isotope composition between two compartments C and D in a soil–plant system can be denoted as Δ_C–D_. The Δ_C–D_ notation describes the difference of the δ-values between C and D, e.g. bulk soil and soil extract, root and shoot, and stem and mature leaves. Due to complex metal cycling within the plant, the isotope difference between two compartments C and D, such as stem and mature leaves, reflects isotope fractionation that is linked to the translocation of metals from C to D, their remobilization from D to C, or their translocation/remobilization from C and D to other compartments E, such as young leaves or grains.

Mass balances and pool size effects are important to consider when interpreting the metal isotope composition of different plant compartments (i.e. pools).^6^  Figure S1 illustrates the pool size effect for roots and shoots. For instance, when the fraction of the metal that is translocated from the roots to the shoots is small, the isotope composition of the root is closer to the isotope composition of the whole plant, and the shoot is isotopically more distinct compared to the whole plant (Fig. S1). If the majority of the metal is transported from roots to shoots, then the shoot shifts towards the isotope composition of the whole plant. Such pool size effects should also be considered in experiments that investigate the isotope fractionation during metal uptake into plants or cells.^21–23^ Simple models of the isotope composition in plants that integrate the equations for isotope mass balances and isotope fractionation factors are presented in the section ‘Special focus on isotope mass balances and modelling in plants’.

### Basics of XAS

XAS is an element-specific spectroscopic technique that provides information about the oxidation state and local geometry of the absorbing element. Samples can be analysed on the micrometre scale with focused X-ray beams, or measured in bulk with a beam in the millimetre range. This technique is almost exclusive to synchrotron facilities due to the required photon flux and spectral resolution,^24^ although bench-top XAS systems have been developed for samples with high metal concentrations.^25^

The XAS spectra are conventionally divided into two regions: X-ray absorption near-edge structure (XANES) and extended X-ray absorption fine structure (EXAFS). The energy region extending from 50 eV below the absorption edge to about 200 eV above the absorption edge is the XANES part of the spectrum. In this part, information about the valence state and the coordination environment of the metal can be retrieved. The EXAFS part of the spectrum is the normalized oscillatory part above the absorption edge to about 800 eV above the adsorption edge and contains information on the local coordination environment of the metal.^14,15^

Traditional shell-by-shell XAS analysis can be used to determine structural parameters for metal complexes, such as the coordination number and the interatomic distances.^14,15^ The shell-by-shell analysis involves filtering by Fourier transformation and back transformations of the EXAFS part. Alternatively, metal species can be quantified by linear combinations of reference spectra using linear least-squares fitting. The library of reference spectra should be as exhaustive as possible and contain reference spectra that are relevant for the environmental system studied. For XANES, this procedure is peculiarly sensitive to the energy calibration and the monochromator resolution. Hence, references and samples should be preferably measured in the same conditions, and a metallic reference foil should be analysed simultaneously downstream of the sample for energy calibration. Linear combination fitting packages are available in XAS data analysis programmes, such as Athena,^26^ XAS viewer,^27^ Sixpack,^28^ and Fastosh.^29^ Detailed discussions on XAS data analyses for environmental systems can be found in.^14,15,30,31^ Shell fitting and linear combination fitting can be performed in parallel to verify if both approaches lead to the same conclusion.

XAS probes the first coordination shells around the target element. This may prevent the distinction between molecules with the same binding element, e.g. glutathione and phytochelatins. The sensitivity to differentiate between similar metal complexes depends on the data quality, which in turn depends on the metal concentration of the sample (for a beamline of equivalent sensitivities). For example, EXAFS allowed to quantify aqueous Ni and Ni-organic acids in the leaves of Ni hyperaccumulating plants with Ni concentrations that ranged from 7.8 to 12.9 mg g^−1^,^32^ whereas on rice shoots containing 20–30 μg g^−1^ Cd, XANES could quantify a Cd–S and a Cd–O pool, without distinction between aqueous Cd and Cd-organic acids.^33^ The sensitivity to distinguish different species also depends on the spectral resolution that is controlled by the monochromator and the detection system. In this respect, the recently developed HERFD-XAS technique is promising (see future directions and conclusions).

### Interaction of metals with minerals and humic acids

**Fig. 1:**
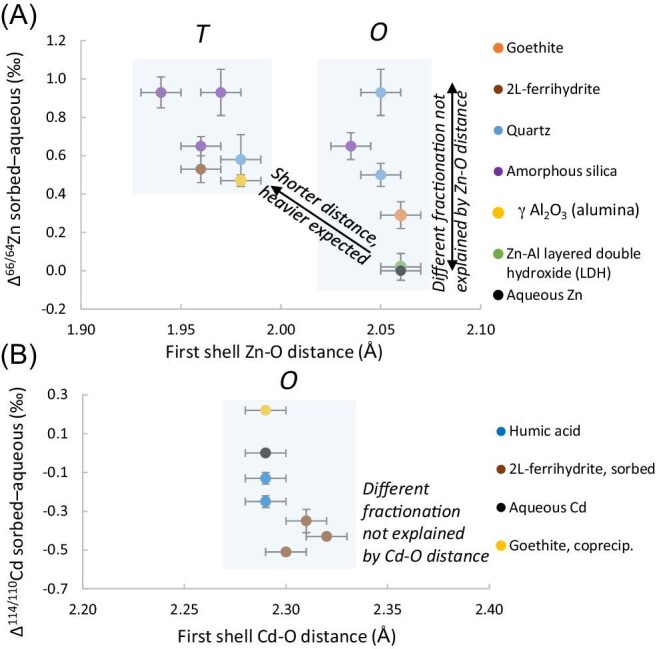
Zn (A) and Cd (B) isotope fractionation during sorption on minerals or complexation with humic acids as a function of the average first shell distance (data from Table 1). For aqueous Zn and Cd, the average distance shown is the one determined by EXAFS at room temperature [2.06 and 2.29 Å, respectively (Table 2)]. T and O denote tetrahedral and octahedral complexes.

A review on equilibrium isotope fractionation during metal sorption and complexation with environmentally relevant surfaces was recently published.^16^ Here, we complement this review with studies that investigated soil surfaces such as minerals and humic acids by coupling XAS and isotope measurements. An overview of these studies is given in Table S1. Several studies have reported Zn isotope fractionation during Zn sorption on mineral surfaces such as hematite, pyrolusite, gibbsite, corundum, goethite, and birnessite.^16^ In these studies, the Zn isotope fractionation at the mineral-solution interface strongly varied. This variation suggests that different mechanisms removed Zn from the solution, such as inner-sphere versus outer-sphere complexation, precipitation, and incorporation. The combined XAS-isotope approach was used to elucidate the relationship between Zn isotope fractionation and Zn sorption mechanisms on goethite and 2-line ferrihydrite,^34^ quartz and amorphous silica,^35^ and γ-alumina.^36^ The metal coordination and average bond length for the sorbed/coprecipitated/complexed metals that were determined by EXAFS are summarized in Table 1 and Fig. 1A. The equilibrium isotope fractionation between the sorbed/coprecipitated/complexed metals and their aqueous form was also determined (e.g. Δ^66/64^Zn_sorbed–aqueous_). Sorbed Zn was either present as an outer-sphere complex, an inner-sphere complex (tetrahedral or octahedral coordination), or as a Zn–LDH surface precipitate. This speciation depended on the type of mineral, Zn surface loading, and ionic strength. Tetrahedral complexes were consistently enriched in heavy isotopes compared to aqueous Zn (Table 1 and Fig. 1A). The extent of this enrichment varied between the substrates. In addition, the mean Zn–O bond length for these tetrahedral complexes ranged between 1.94 and 1.98 Å (Table 1). For comparison, the average bond length for aqueous Zn in octahedral coordination (Zn–O) was 2.06 ± 0.01 Å (Table 1). The difference in coordination and in bond length was proposed to drive the observed isotope fractionation towards heavier isotopes in tetrahedrally sorbed Zn (Fig. 1A). In octahedral Zn complexes/precipitates, a variable isotope fractionation with either no significant or a positive fractionation was observed despite the similar coordination and bond length (Table 1 and Fig. 1A).

**Table 1.** Comparison of isotope fractionation between sorbed/coprecipitated/complexed and aqueous metal and first shell parameters for the sorbed/complexed metal

	Conditions				First shell parameters^a^				
Sorbing or complexing phase	Ionic strength	Surface coverage	Δ^66/64^Zn_sorbed–aqueous_ (‰)	±	Zn–O distance (A)	±	Type	Coord.	Refs
Goethite			0.29	0.06	2.06	0	Sorbed, inner S	O	[29]
Zn-Al layered double hydroxide (LDH)			0.02	0.03	2.06	0	Inner, coprecip.	O	[31]
Quartz	Low (0.004 M)	Low (Γ = 0.5 μmol m^−2^)	0.50	0.07	2.05	0	Sorbed, outer S.	O	[30]
Quartz	High (0.1 M)	Low (Γ<0.2 μmol m^−2^)	0.93	0.07	2.05	0	Sorbed, inner S	O	[30]
Amorphous silica	Low (0.004 M)	Interm. (Γ∼0.3 μmol m^−2^)	0.65	0.06	2.03 to 2.04		Sorbed, inner S	T + O	[30]
Quartz	High (0.1 M)	High (Γ = 1.3 μmol m^−2^)	0.58	0.12	1.98	0	Sorbed, inner S	T	[30]
γ-Al_2_O_3_ (alumina)			0.47	0.13	1.98	0	Sorbed, inner S	T	[31]
Amorphous silica	High (0.1 M)	Low (Γ < 0.2 μmol m^−2^)	0.94	0.07	1.97	0	Sorbed, inner S	T	[30]
Amorphous silica	Low (0.004 M)	High (Γ > 0.7 μmol m^−2^)	0.65	0.12	1.96	0	Sorbed, inner S	T	[30]
2L-ferrihydrite			0.53	0.05	1.96	0	Sorbed, inner S	T	[29]
Amorphous silica	High (0.1 M)	High (Γ = 1.1–1.4 μmol m^−2^)	0.93	0.08	1.94	0	Sorbed, inner S	T	[30]
			**Δ^114/110^Cd _sorbed–aqueous_**		**Cd–O distance**				
	**Conditions**		**(‰)**	**±**	**(A)**	**±**	**Type**	**Coord.**	
Humic acid	High (0.1 M)		–0.13	0.03	2.29	0	Complexed, inner S.	O	[34]
	Low (0.001 M)		–0.25	0.03	2.29^b^	0	Complexed, outer S.	O	[34]
Goethite			0.22	0.01	2.29	0.1	Inner, coprecip.	O	[32]
2L-ferrihydrite			–0.55	0.10	2.28–2.32^c^		Sorbed, inner S	O	[32]

Samples are ordered by decreasing interatomic distance.

^a^Average interatomic distance, type of interaction and coordination type (tetrahedral T or octahedral O).

^b^Data for aqueous Cd, from reference ^109^.

^c^Cumulant analysis suggests that the octahedron is highly disordered. n.d.: not determined.

The combined XAS-isotope approach has also been used to investigate Cd sorption on ferrihydrite.^37^ Preferentially light Cd isotopes adsorbed to this mineral (Δ^114^Cd_sorbed–aqueous_ = –0.51 to –0.55‰ (Table 1 and Fig. 1B). Here, octahedral Cd–O complexes with a mean Cd–O bond distance of 2.28–2.32 Å were observed with EXAFS. This bond length was comparable to the Cd–O distance of the aqueous metal [2.29 Å (Table 2)]. Moreover, these complexes were highly distorted based on a third cumulant model.^38^ This distortion may be the main driver for the enrichment of heavy isotopes in the aqueous phase and may also explain the enrichment of heavy isotopes in soil solution and phytoavailable soil pools (see section ‘The soil–soil solution–plant interfaces’, Fig. 3A). Unlike Cd adsorption to ferrihydrite, coprecipitation of Cd with goethite led to an enrichment of heavy isotopes in the solid phase (Δ^114/110^Cd_incorperated–aqueous_ = +0.22‰ ) (Table 1 and Fig. 1B). The average Cd–O distance in the precipitate was the same as in the aqueous metal (2.29 Å, Table 2 and Fig. 1B).

**Table 2.** Average Zn–O and Cd–O distance and the method used for aqueous Zn and Cd

Zn–O distance	Method	References
2.06 ± 0.02	EXAFS, room temperature	[115]
2.06	EXAFS, room temperature	[36]
2.07	EXAFS, room temperature	[35]
Cd–O distance		
2.33	Density functional theory	[42]
2.29	X-ray diffraction	[116]
2.34	Unbiased molecular dynamics	[117]
2.29^a^	EXAFS, 17°C	[118]
2.31	EXAFS, 20°K	[49]

^a^This distance was obtained in similar conditions (EXAFS at room temperature) as the one obtained on sorbed/coprecipitated/complexed species. This distance is thus recommended to use for comparison.

Cadmium complexation with humic acids has also been studied by the combined XAS-isotope approach.^39^ Regardless of the chemical conditions in the solution, such as pH and ionic strength, the Δ^114/110^Cd_HA–solution_ was negative (Table 1). At high ionic strength, Cd formed inner-sphere complexes in which Cd was hexacoordinated with carboxyl groups (Δ_HA–Cd(aq)_ = –0.13‰). At low ionic strength, non-specific Cd binding driven by electrostatic attraction (outer-sphere complex) dominated the Cd binding to humic acids. This electrostatic attraction induced a stronger isotope fractionation (Δ_HA–Cd(aq)_ = –0.25‰) than specific Cd binding by carboxyl groups. The average Cd–O bond length of the inner-sphere complex was 2.29 Å and did not significantly differ from aqueous Cd (2.29 Å). More information on the local environment of Cd in the hydrated form as well as on the inner and outer-sphere complexes would be necessary to progress on the interpretation of the isotope fractionation that is induced by Cd sorption to minerals and complexation with humic acids.

As for Cd, light Ni isotopes preferentially sorbed on ferrihydrite (Δ^60/58^Ni_sorbed–aqueous_ = –0.35 ± 0.08‰). In addition, the Ni isotope fractionation was stronger for goethite (Δ^60/58^Ni_sorbed–aqueous_ = –0.77 ± 0.23‰) compared to ferrihydrite, despite similar octahedral coordination and bond length for aqueous and sorbed Ni between these two minerals.^40^ The only difference in Ni speciation between goethite and ferrihydrite was a lower Ni–Fe coordination number in the second shell for goethite. It was therefore proposed that this difference in the second shell coordination may influence the distortion of the Ni–O coordination sphere and control the difference in isotope fractionation.

These first applications of the combined XAS-isotope approach on single phases revealed that the first shell parameters obtained by XAS (coordination and average bond length) are in several cases insufficient to explain the isotope fractionation between the sorbed metal and the hydrated metal ion. Several additional parameters can affect the metal isotope fractionation, including distortion of the metal octahedron after sorption,^37^ higher coordination shells in the case of inner-sphere complexes,^35,40^ and electrostatic interactions in the case of outer-sphere complexes.^39^ More data is needed to improve the interpretation of isotope fractionation in single phases. In particular, data on electrostatic interactions could be provided by theoretical calculations, as done for binding interactions for Zn^41^ and Cd.^42,43^

### Soils: pedological processes and source tracing

**Fig. 2:**
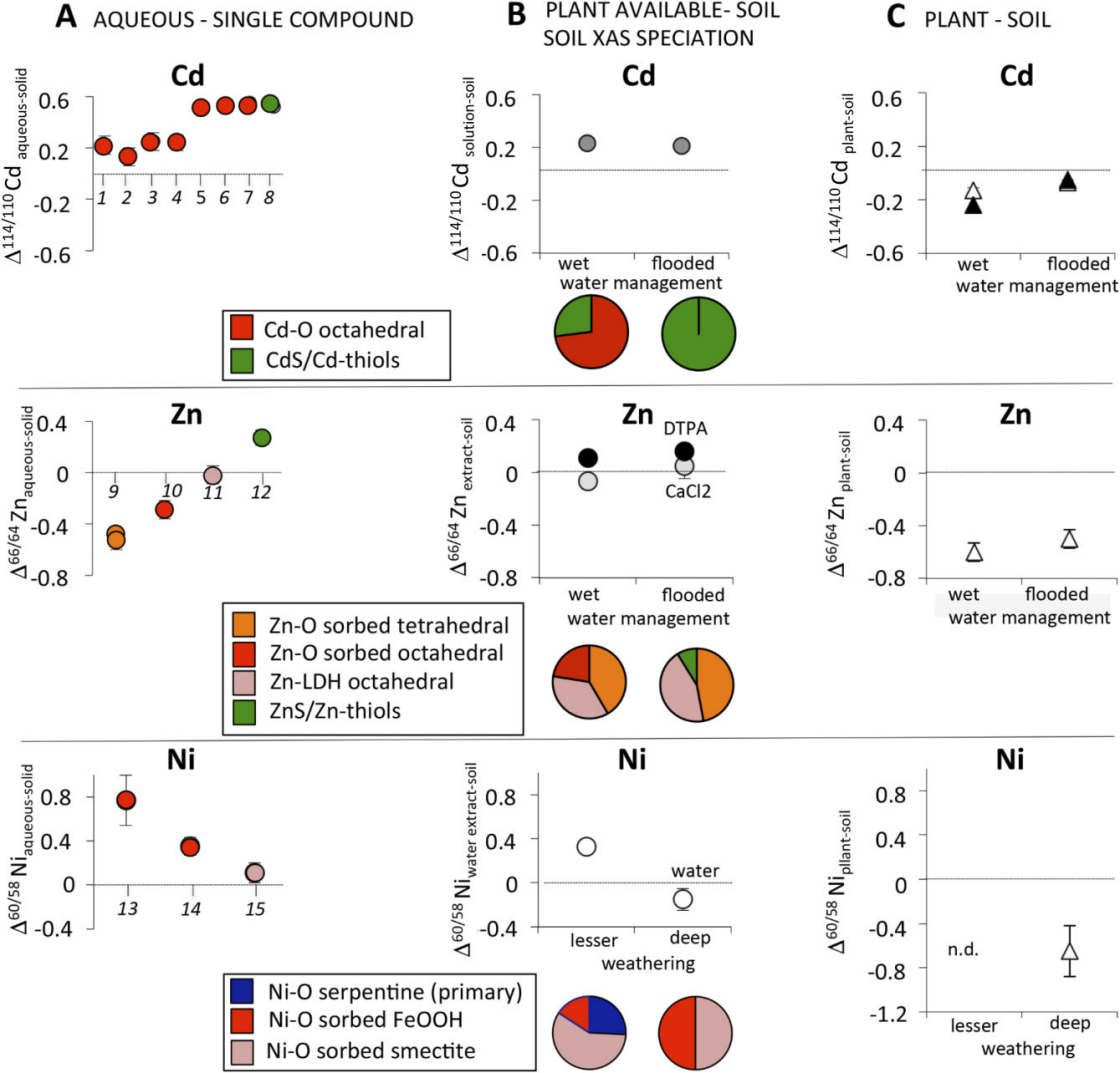
Isotope fractionation and speciation for Cd, Zn, and Ni in systems of increasing complexity. This figure shows how the information on metal speciation and isotope fractionation for single phases (minerals, humic acids), combined with metal speciation in the soil, can help to interpret differences in isotope composition between the plant, the soil, and the soil solution/soil extract. (A) Isotope fractionation between aqueous metals and sorbed/complexes species. 1: coprecipitation with goethite;^37^ 2: humic acid complex, inner sphere^39^; 3: humic acid complex, outer sphere^39^; 4: sorption on birnessite;^53^ 5–7: sorption on goethite, hematite, ferrihydrite^37^ 8: CdS precipitate in freshwater^50^; 9: sorption, tetrahedral coordination;^34,36^ 10: sorption, octahedral coordination^34^; 11: Zn–LDH^34^; 12: ZnS precipitate^48^; 13, 14: sorption on goethite and ferrihydrite, respectively (octahedral coordination)^40^; 15: sorption on smectite (coordination not specified).^119^ (B) Soil XAS speciation and differences in isotope composition between plant-available metal and soil.^18,32,47,49^ Plant-available metal: given by metal in solution (Cd) or soil extracts with DTPA, CaCl_2_ (Zn), and water (Ni). (C) Difference in isotope composition between plant and soil (references are the same as for B). n.d.: not determined. For Cd, two plant genotypes were studied: the excluder (empty symbols) and non-excluder (black symbols) rice.

Nickel isotope fractionation and XAS were used to study weathering processes in a lateritic profile.^44^ By studying the various layers of the profile (regolith, saprock, saprolite, and laterite), a progressive replacement of Ni-bearing serpentines at the base of the saprolite was shown. This replacement was driven by Ni-sorbed iron oxides while this change in Ni speciation was accompanied by a depletion in heavy isotopes. The same trend was observed by Zelano et al. (2020)^32^ in two Ni-rich ultramafic soils at different stages of weathering. The less weathered soil, which contained Ni-serpentine and Ni sorbed on clays and goethite, was isotopically heavier than the more weathered soil (δ^60/58^Ni = –0.01 ± –0.05‰ and –0.17 ± –0.01‰, respectively (Fig. 2B). This strongly weathered soil had no Ni-serpentine left, but most Ni was associated with goethite. This observation was consistent with the preferential incorporation of light Ni-isotopes into Fe-oxides^16,40^ (Fig. 2A) and with the enrichment of heavy Ni isotopes in the local groundwater of the lateritic soils.^44^

The combined XAS-isotope approach served to investigate an agricultural and a forest soil that were located next to a Zn smelter.^45^ In both soils, lighter Zn isotope compositions in the lower horizons (δ^66/64^ Zn = +0.31 ± 0.38‰) were interpreted as being representative of the local geochemical background. In contrast, heavier isotope compositions towards the surface of the two soils were related to inputs of anthropogenic Zn. Indeed, the smelter slags were strongly enriched in heavy Zn (δ^66/64^ Zn = +0.81 ± 0.20‰) and contained franklinite (ZnFe_2_O_4_). This mineral was present in the two topsoils along with secondary phases (Zn-humate, Zn-illite, Zn/Al–LDH, and Zn-talc). However, in the forest soil, an enrichment of light Zn isotopes in the top layer (2.5 cm) suggested either additional anthropogenic sources that were enriched in light Zn or post-depositional processes. Suggested anthropogenic sources were Zn-sulphides and Zn-sulphates that were emitted from the chimney of the smelter. Zn-bearing mineral precipitation, complexation of Zn by organic matter, Zn plant uptake, and litter fall may have changed the isotope composition in the soil profile after the Zn deposition.

These studies on soils show that metal isotope compositions can be a good indicator for weathering processes and anthropogenic sources in the soil. The XAS-isotope approach assisted in the description of pedological processes as well as in the disentanglement of sources and processes that determine the distribution of metals in anthropogenically impacted soils.

### The soil–soil solution–plant interfaces

The soil-to-plant transfer of metals consists of several chemical (dissolution, desorption, chelation), physical (diffusion, mass flow), and biological (transmembrane transport) processes that potentially control their isotope fractionation.^46^ Several of these processes are linked to changes in metal speciation. For example, changes in Zn speciation in the bulk soil can affect the Zn isotope fractionation during soil-to-plant transfer, as shown in soils from an urban wetland.^47^ In these metal-contaminated soils, soil flooding increased the proportion of Zn bound to S groups by 9% and increased the isotope fractionation between the plant and the bulk soil (Δ^66^Zn_plant–soil_) from –0.58 to –0.48‰ (Table 3, Fig. 2C). This isotope shift can be partly ascribed to the precipitation of light Zn with sulphides in the soil,^48^ which enhanced the transfer of heavy isotopes from soil to plant.

**Table 3.** Summary of difference in isotope composition (Δ_C–D_ in ‰) for different plant-soil systems discussed in this review

Plant and growing conditions		Metal concentration (in mg kg_−1_ soil or μM or μg L_−1_ solution)	Δsoil solution–soil or Δsoil extract–soil	Δwhole plant–soil	Δwhole plant–soil extract	Δwhole plant–nutrient or soil solution	Δshoot–root	Δleaf–root	Δleaves–stem	Δgrain–shoot	Δdead leaves–fresh leaves	References
Tomato, Fe sufficient	Cu	1 μM or 63.5 μg L^−1^				– 1.05	1.03		–0.37			[17]
Tomato, Fe deficient	Cu	1 μM or 63.5 μg L^−1^				–0.99	0.66		–0.34			[17]
Oat, Fe sufficient	Cu	1 μM or 63.5 μg L^−1^				–0.20	–0.04		–0.06			[17]
Oat, Fe deficient	Cu	1 μM or 63.5 μg L^−1^				–0.11	0.04		0.00			[17]
Reed canary grass^1^, rarely flooded zone	Zn	2129 mg kg^−1^	–0.07	–0.58	–0.51		–0.80	–0.43	0.40			[18]
Broadleaf cattail^2^, frequently flooded zone	Zn	2089 mg kg^−1^	0.05	–0.48	–0.53		–0.40	–0.35	0.16			[42]
Rice, non-excluder^a^, flooded cond., flowering	Cd	15 mg kg^−1^	0.17–0.21	–0.05		–0.27	0.09					[44]
Rice, excluder^b^, flooded cond., flowering	Cd	15 mg kg^−1^	0.17–0.21	–0.07		–0.24	0.19					[44]
Rice, non-excluder^a^, wet cond., flowering	Cd	15 mg kg^−1^	0.17–0.21	–0.24		–0.43	-0.02					[44]
Rice, excluder^b^, wet cond., flowering	Cd	15 mg kg^−1^	0.17–0.21	–0.13	–0.27	–0.30	0.16					[44]
Rice, excluder^b^, wet cond., maturity	Cd	15 mg kg^−1^		–0.07	–0.32		0.28			0.66		[44]
Ni hyperaccumulating tree^3^, 1-year old	Ni	6114 mg kg^−1^	–0.02 to 0.15	–0.80 to –0.67				–0.51 to –0.28				[27]
Ni hyperaccumulating tree^3^, 3-year old	Ni	2939 mg kg^−1^	–0.51 to –0.33					0.26–0.40				[27]
*Macadamia integrifolia* (tree), humid soil	K	28700 mg kg^−1^ (subsoil)	–0.09								–0.77	[79]
*Pennisetum setaceum* (grass), humid soil	K	8700 mg kg^−1^ (topsoil)	0.83				0.08	0.11	0.17		–0.60	[79]
*Prosopis chilensis* (tree), arid soil	K	1700 mg kg^−1^ (subsoil)	0.72								–0.84	[79]
*Cenchrus ciliaris* (grass), arid soil	K	1800 mg kg^−1^ (topsoil)	0.68				0.10	0.22	0.34		–0.82	[79]

(A) positive value indicates that compartment (C) is enriched in heavy isotopes compared to compartment (B).

^a^Cultivar TCM-213 (dysfunctional vacuolar transporter HMA3 in roots).

^b^Cultivar taichung-65 (HMA3 functional). ^1^*Phalaris arundinacea*, ^2^*Typha latifolia*, ^3^*Rinorea cf. bengalensis*. For Tl: values given as epsilon = delta × 10 in the article.

Similarly, in a Cd-spiked rice paddy soil, XAS showed that in non-flooded conditions, most of the Cd in the soil was bound to O donors (87%). The binding of Cd to O donors was ascribed to soil organic matter or Fe/Mn hydroxides, while the minority was bound to S donors such as thiol groups (13%).^49^ After flooding, Cd was fully bound to inorganic S (sulphides) and organic S (thiol groups). The Δ^114/110^Cd_plant-bulk.soil_ increased from –0.13‰ in non-flooded conditions to –0.07‰ in flooded conditions^49^ (Table 3, Fig. 2C). This is in line with light Cd isotopes precipitation with sulphides.^50^ In addition to Cd precipitation with sulphides, the soil-to-plant transfer of Cd can decrease upon flooding because of changes in soil Eh and pH.^51^ Changes in Eh can increase soil pH, which typically leads to an increased sorption of Cd to, e.g. metal oxides and organic matter.^52^ This sorption step usually occurs before the formation of Cd sulphides and should induce a positive shift of the isotope composition in the soil solution.^37,53^ Note that the isotope shifts measured for Cd sorption in Fe oxyhydroxides^37^ are actually similar to the ones measured for CdS precipitation^50^ (Fig. 2A). However, no significant changes in Δ^114/110^Cd_soil solution–soil_ were detected through time.^49^ This lack of detection was related to the fast removal of Cd from the soil solution to the soil due to increasing pH. To yield sufficient Cd in the soil solution for isotope analyses, samples had thus to be pooled. This pooling did not allow to record changes in Δ^114/110^Cd_soil solution–soil_ during soil flooding, although a difference in Δ^114/110^Cd_plant–soil_ was observed between flooded and non-flooded conditions (see above). Hence, more studies are needed to evaluate the intermediate effects of flooding on the Cd isotope composition of the soil solution.

Generally, the Δ_plant–phytoavailable.pool_ is more meaningful than the Δ_plant–soil_ to determine the isotope fractionation during plant uptake, as only a fraction of the total stock of metals in a soil is available to plants. The determination of Δ_plant–phytoavailable pool_ requires measurements of the isotope composition of the entire plant and of the phytoavailable metal pool.^54^ The latter can be estimated by using salts,^18^ diluted acids (HCl,^55^), and resin-based methods such as DGT.^56,57^ However, the concentration and isotope composition of the extracted soil pools differ depending on the method used.^58^ This has also been shown for phytoavailable Zn pools in contaminated soil that were extracted by CaCl_2_, and DTPA.^47^ The DTPA extracted up to 50 times more Zn compared to CaCl_2_, and the Zn isotope was 0.11–0.18‰ heavier in the DTPA compared to the CaCl_2_ soil pool. Using CaCl_2_, Ca exchanges with weakly bound Zn on negatively charged binding sites of the soil (i.e. outer-sphere complexes), while Cl may provide a complexation anion that potentially increases the Zn solubility.^59^ In contrast, DTPA chelates and thereby extracts Zn from weak but also stronger binding sites (i.e. outer and inner-sphere complexes). The heavier isotope composition in the DTPA extract corresponds with isotope fractionation factors for Zn-chelating organic ligands that are similar to DTPA.^60^ This example illustrates that the extracted phytoavailable soil pool is determined by the type of extractant and is therefore operationally defined.^59,61^ Nevertheless, such extracts can provide useful insights if the isotope fractionation during uptake is compared between different factors and treatments, such as distinct soils,^19,58^ plant species/cultivars,^47,62^ and distinct soil management strategies.^63^ A further option to collect information on the phytoavailable pool is to sample the soil solution. This method allows to non-destructively detect temporal changes in metal concentrations and isotope compositions in soil–plant systems.^49^ Together, the use of phytoavailable soil pools to determine the isotope fractionation during plant uptake requires that the resulting isotope composition of the phytoavailable pool is discussed regarding its operational characteristics.

Combining chemical extractions with XAS and isotopes can provide additional insights on the metal exchangeable pools. For instance, in the Zn-contaminated urban wetland soil mentioned above, the Δ^66^Zn_CaCl2 extract–soil_ was slightly lower in the drier area (–0.07‰) compared to the frequently flooded zone (0.05‰) (Fig. 2B).^18,47^ This enrichment of heavy Zn isotopes in the CaCl_2_ extract in the flooded zone is consistent with the preferential incorporation of light Zn isotopes in sulphides as mentioned above. In the soil of the drier area, Zn was bound to a mixture of tetrahedral and octahedral Zn–O species that preferentially sorbed heavier Zn isotopes and depleted the extract in lighter Zn isotopes. These findings were in agreement with experimental results conducted with single mineral phases (Table 1). Additionally, a positive relationship was found for different soil size fractions between the proportion of tetrahedral Zn and the Zn isotope composition. These results were also in line with experimental results on single mineral phases (Table 1). The DTPA extract in the dry soils accounted for 18% of the total Zn in the soils and was enriched in heavy isotopes compared to the initial bulk soils.^18^ After DTPA extraction, the fraction of tetrahedral Zn species in the bulk soil significantly declined, indicating that DTPA extracted primarily tetrahedral Zn complexes, together with some weakly bound octahedral Zn (1% of total soil Zn).

**Fig. 3:**
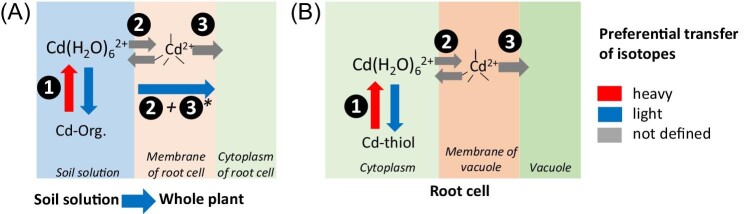
**A**. Cd isotope fractionation from the soil solution to the plant favours light isotopes (bottom of A) and is governed by several processes. (1) In the soil solution, complexation of Cd to humic acids favours light Cd isotopes, which results in enrichment of the hydrated Cd species in heavy isotopes.^39^ (2) Binding of Cd^2+^ to the metal ion-binding site of a membrane protein. This binding step requires the replacement of the water molecules by donor atoms at the binding site of the membrane transport protein. (3) Changes in the conformation of the membrane protein drive the release of Cd^2+^ into the cytoplasm. *The transport by, e.g. TcNRAMP5 (steps 2 + 3) favours light Cd isotopes.^73^ (B) Cd isotope fractionation from the root symplast to the vacuole. This step is governed by similar processes than in (A): The isotope fractionation is driven by Cd binding to thiol-containing ligands (1), dehydration of Cd (2), and cross-membrane transport (3). Note that the isotope ratio in the root is also affected by Cd binding in the vacuole, Cd efflux out of the vacuole (not shown), and transfer to shoots (see Fig. 4). Step 2 in both figures illustrates the hypothesis of isotope equilibrium between the metal in aqueous phase and the metal bound to the transporter binding site.^11,72^ Two other scenarios for fractionation during metal transport have been proposed: (i) The fractionation induced by the membrane transport essentially reflects the fractionation between the free (or alternatively, desolvated) metal and the complexed metal in solution (A) or in the cytosol (B) (e.g. references ^11, 22^, and ^23^). (ii) Alternatively, kinetic isotope effects might drive the fractionation^6,11^ as the transport process is unidirectional. Note that the metal transport pathway in the transporter is complex. The metal cation may enter the transporter through an electronegative funnel or cavity before reaching the intramembraneous ion-binding site.^120,121^

The combined XAS-isotope approach was also applied for Ni soil extracts. Zelano *et al.*^32^ performed water extractions on two Ni-rich ultramafic soils at different stages of weathering to evaluate the isotope composition of the phytoavailable pool. Water-extractable Ni was heavier than the bulk soil at a less advanced stage of weathering, but lighter in the deeply weathered soil (Figs. 2–3b). Based on Ni speciation in the bulk soil (see section ‘Soils: pedological processes and source tracing’), the distinct isotope fractionation was interpreted by losses of soluble and heavy Ni during weathering of Ni-serpentine. These examples on Zn and Ni show that the combined XAS-isotope approach provides additional information on the local structure of the extracted metal pools in the soils.

In addition to metal speciation in the solid soil phase, metal speciation in the soil solution is another parameter that contributes to the isotope fractionation between soils and plants.^46^ During metal uptake by transporter proteins, in most cases, the species transported is the uncomplexed metal.^64^ The speciation in solution affects the isotope composition of this uncomplexed metal pool, as shown in single phases (see section ‘Interaction with minerals and humic acids’). Hence, it likely affects the isotope composition of the plant, as shown by Wei et al. (2018)^65^ for Cd-accumulating plants that were exposed to hydroponic nutrient solutions with and without the chelator EDTA. Studying metal speciation in the soil solution by XAS is highly challenging because of the low metal concentrations in the soil solution. Alternatively, speciation modelling^49,66^ and chromatographic ICP-MS techniques^67^ facilitate the interpretation of the role of metal speciation in solution on isotope fractionation during plant uptake.

Besides the metal speciation in the solid soil phases and in the soil solution, metal sorption onto the root apoplast can also induce isotope fractionation.^18,47,68,69^ Aucour *et al.*^47^ extracted Zn from the roots of a wetland plant *(Typha latifolia)* with HCl. This extraction targets the iron plaque on the roots that is typically formed in wetland plants. The extraction of the iron plaque led to a loss of Zn that was bound tetra and octahedrally to O donors (each 20%), while Zn bound to S donors proportionally increased. However, the isotope composition of the extracted Zn was not distinguishable from the Zn in the bulk root. In addition, based on Zn mass balances, the changes in Zn speciation were too large, which suggests that artefacts may have been created during the Zn extraction from the root.^47^ This study showed that chemical extraction and the combined XAS-isotope approach together could provide information on the functional role of the apoplastic Zn pools for the plant. However, it is important to keep the intracellular compartments of the root intact during the root extractions.

Finally, the plant uptake itself through membrane transport can also fractionate metal isotopes (Fig. 3A). As explained above, complexed metals and hydrated metal ions (also called aquo complexes) are present in the soil solution. Prior to cross-membrane transport, metals may associate and dissociate from complexing ligands in the soil solution (step 1 in Fig. 3A) and the uptake of the metals as hydrated or desolvated metal ions may thus induce an isotope fractionation. Then, water molecules of the hydration sphere are replaced by ligands from the metal binding site of the membrane transporter. Exemplified for Cd, the six water molecules of the hydrated Cd ion should be exchanged by four ligands from amino acids of the membrane protein (step 2 in Fig. 3A).^70^ This step prior to the actual cross-membrane transport may be equilibrium or kinetically controlled and may thereby favour light and/or heavy isotopes.^54^ A study that investigated Zn uptake into marine diatoms suggested a switch from kinetic to equilibrium control as the Zn uptake rate declines.^11^ Equilibrium isotope effect between hydrated metal ion and binding site may thus prevail when the transport rate is low and kinetic processes may be more dominant when the transport rate is high. The overall extent of fractionation by membrane transporters (step 2 + 3 in Fig. 3A) thus depends on the type of membrane protein, including its binding sites and transport rates.^11,71,72^ In addition, in redox-sensitive metals like Cu and Fe, chemical reduction prior to cross-membrane transport may also contribute to isotope fractionation^72^ (not shown in Fig. 3A). Such a reduction step would favour light isotopes.

The complex metal isotope fractionation during cross-membrane transport can be approached by using unicellular model organisms. The gene *TcNRAMP5* that is involved into Cd uptake in *Theobroma cacao* was expressed in yeast. This experiment showed that the membrane transporter TcNRAMP5 favoured light Cd isotopes^73^ (step 2 + 3 in Fig. 3A). Similarly, Cadiou *et al.*^72^ showed that the preferential uptake of light Cu isotopes in yeast was mainly due to a high affinity transporter and modulated by the reductases that convert Cu(II) to Cu(I) prior to membrane transport. Such experiments are useful to determine the metal isotope fractionation that is induced by membrane proteins that control metal translocation in plants.

This overview of the soil–soil solution–plant interface shows the multiplicity of isotope fractionation steps that occur during the journey of the metal from the soil to the plant. It further showed that the XAS-isotope approach in combination with chemical extractions helped to describe the relationship between changing environmental conditions, metal speciation, and isotope fractionation, and to decompose some of the various isotope fractionation steps from the bulk soil to the plant.

### Root to shoot translocation

The fraction of the metal that is not retained in roots by, e.g. sequestration in vacuoles and/or binding to cell walls can be translocated to shoots after xylem loading. XAS allows to elucidate metal storage mechanisms while isotope fractionation allows to observe changes in metal speciation and transport processes in the roots (through, e.g. diffusion, membrane transport). The XAS-isotope approach was used to study Cu in plants that were grown in hydroponics with a high Cu(II) supply.^17^ The Cu isotope fractionation between root and shoot (Δ^65^Cu_shoot–root_) differed between Fe-strategy II plants (oat: –0.04 to 0.04‰) and Fe-strategy I plants (tomato: 0.66–1.03‰). However, Cu speciation was similar in the roots of both plants, with Cu(I)–S as predominant species (58–87% of total Cu) and Cu(II)–O/N as minor species. For tomato, the authors proposed that after the uptake of Cu as Cu(I), it was detoxified through the formation of Cu(I) thiol clusters. With root-to-shoot translocation, Cu(I) was oxidized to Cu(II) species such as Cu(II)-nicotianamine, which led to an enrichment of heavy isotopes in the shoots. In oat, the absence of Cu isotope fractionation between root and shoot suggested that Cu translocation did not involve redox reactions.

For Zn, Aucour *et al.*^18^ observed a retention of heavier Zn isotopes in the roots of *Phalaris arundinacea*, which resulted in a Δ^66/64^Zn_shoot–root_ value of –0.80‰ (Table 3). The combination of bulk EXAFS on roots and isotope measurements before and after HCl extraction suggested that Zn was mainly present as octahedral complexes in the vacuoles (65%), and secondarily as tetrahedral complexes in cell walls (35%). The authors concluded that the Zn enrichment of heavier isotopes in roots could be due to membrane transporters directing Zn to the vacuoles and indirectly to the preferential export of light isotopes into the xylem. A similar proportion of tetrahedral Zn (40%) was found in the roots of *T. latifolia*.^47^ For this latter species, a lower retention of heavier Zn isotopes was observed in roots, with Δ^66/64^Zn_shoot–root_ of about. –0.40‰ (Table 3).^47^ This observation was explained by the presence of a minor fraction of Zn bound to thiol ligands in the roots of this plant, in addition to vacuolar Zn−OAs and Zn−cell wall complexes. Based on *ab initio* calculations, lighter Zn isotopes bind to thiol groups at equilibrium.^41^ Therefore, Cd–S complexes may have contributed to a lighter Zn isotope composition in the roots of *T. latifolia* compared to *P. arundinacea*. Together, the observed Zn speciation and isotope difference between roots and shoots suggested that a combination of membrane transporter and ligand effects controlled the transport of Zn from root to shoot.

**Fig. 4:**
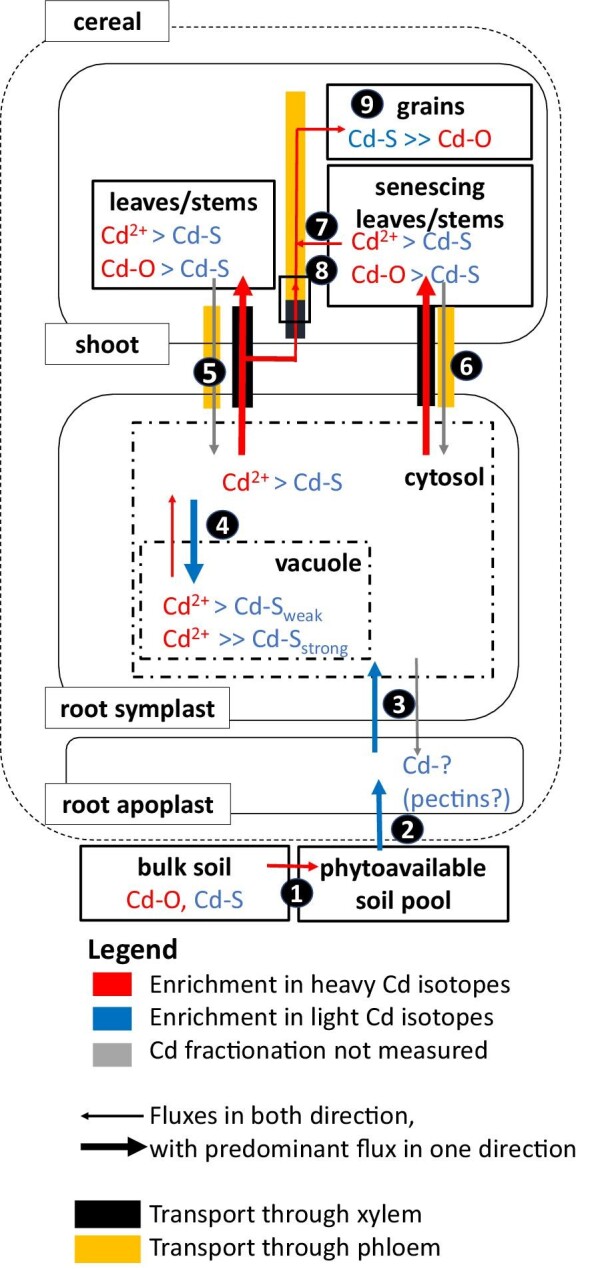
Proposition of Cd fluxes, isotope fractionation, and speciation within a cereal. Size of the boxes is not proportional to Cd fractions in plants. (1) Part of the Cd in the soil is phytoavailable.^46^ (2) Light Cd is adsorbed to the root apoplast.^49,85^ (3) The transfer of Cd^2+^ into the root cytosol favours light isotopes, most likely through cross-membrane transport facilitated by, e.g. the protein NRAMP5.^73^ Backward fluxes from the cytosol into the apoplast are small.^105^ (4) In the root symplast, genetic approaches combined with XAS and isotope analyses revealed that light isotopes are sequestrated in vacuoles, likely through cross-membrane transport by HMA3^122^ and/or chelation of Cd by strongly chelating thiols within the vacuole.^43,49^ (5) Preferentially heavy Cd isotopes are transported from root to shoot (stem and leaves) via the xylem, probably because of heavy Cd in the root cytosol (see 4). (6) A fraction of Cd can be transported from shoot to root and from leaves/stems to grains via the phloem, but this fraction is negligible.^123–125^ (7) In stems and leaves, Cd is stored as Cd–S (thiols) and Cd–O, the proportion varies with growth stage.^33^ Cd–O may represent Cd bound to pectins in the shoot apoplast and/or Cd bound to organic acids in vacuoles. Cd remobilization from phloem source (e.g. senescing stems and leaves) to phloem sink tissues (e.g. grains) favours heavy isotopes.^33,85^ (8) Alternatively, Cd can be directly transferred from xylem to phloem in the nodes and then into the grains. Combined isotope and gene expression analyses suggest that this transfer, in part mediated by HMA2 and LCT1, favours heavy Cd for xylem to phloem transfer,^85^ while Cd is mostly bound to S in nodes.^33^ (9) The grain is a phloem sink where the majority of Cd is complexed to Cd–S. Figure layout based on references ^126^ and ^20^.

An opposing isotope fractionation to Zn was observed for Cd in gramineous plants,^74^ although the translocation pathways of these two metals are considered to be similar.^75,76^ The opposing isotope fractionation of Zn and Cd in cereals suggests that, in the experimental conditions tested, diffusion was not the major process. Otherwise, a similar direction of fractionation would be observed for both metals. Alternatively, the distinct affinity of Cd and Zn for O and S donor atoms of organic ligands may cause opposing isotope fractionation.^74^ This hypothesis may apply not only to complexation by ligands in the various plant compartments,^43,60,77^ but also to the binding of Cd to high affinity sites of membrane proteins (Fig. 3A). To investigate if thiol-containing ligands are involved in the sequestration of light Cd into the vacuole, rice accessions with a functional and dysfunctional vacuolar-membrane transporter HMA3 were used.^49^ The isotope fractionation between root and shoot (Δ^114/110^Cd _shoot–root_) was smaller in a rice accession with non-functional HMA3 (–0.02 to 0.08‰) compared to accessions with functional HMA3 (0.16–0.19‰, Table 3). The data thus suggested that Δ^114/110^Cd_shoot–root_ is strongly determined by vacuolar sequestration of Cd. XANES revealed that the Cd in the roots was fully bound to S in both rice accessions. Vacuolar sequestration likely includes transport through the tonoplast by HMA3 and mechanisms that keep Cd in the vacuole (i.e. to sequester it), such as the chelation of Cd by phytochelatins.^78^ Different Cd-thiol species are difficult to identify with XANES and EXAFS. However, *ab initio* calculations predict that chelating ligands such as phytochelatins bind lighter Cd isotopes than ligands with a single thiol group such as glutathione.^43^ Hence, after the transport of Cd into the vacuole, lighter Cd isotopes may be preferentially kept in the vacuole as stable Cd thiolates; thereby, heavier isotopes may be transported from the vacuole back into cytosol, loaded into the xylem, and translocated to the shoot (Fig. 4). The sequestration of lighter isotopes in the vacuole would progressively remove light isotopes during the radial transport towards the xylem, leading to a Rayleigh-like fractionation.^49^ In another study, the overexpression of *OsHMA3* led to a stronger retention of light isotopes in the roots (Δ^114/110^Cd _shoot–root_ = 0.08‰) compared to the wild type rice (Δ^114/110^Cd _shoot–root_ = –0.06‰).^79^ These results confirm that HMA3 is involved in the retention of light Cd isotopes in vacuoles of rice roots but do not reject the hypothesis that coupled HMA3 import and chelation by stable Cd thiolates in the vacuole cause the observed isotope fractionation. Furthermore, a role of the protein CAL1 in root–shoot translocation and isotope fractionation has been reported in rice.^69^ The Cd-CAL1 complex is thought to be secreted from root cells into the xylem and to involve Cd binding by thiol groups.^80^ Higher expression of *CAL1* corresponded to a shift towards the translocation of lighter isotopes from root to shoot.^69^ These observations are consistent with preferential binding of light Cd isotopes to thiol groups in organic ligands.^43^ Together, the studies on Cd suggest that membrane transport and binding of Cd to organic ligands may be the main drivers of isotope fractionation from root to shoot, but it is challenging to disentangle their respective role.

To disentangle isotope effects from membrane transport and organic ligands, knowledge of isotope fractionation during membrane transport is required. Prior to cross-membrane transport as cations, metals may dissociate from organic ligands since they are supposed to be complexed in the cytosol due to its chemical composition and alkaline pH^64,81^ (Fig. 3B). The membrane transport itself generally involves several steps that potentially fractionate Zn and Cd isotopes. Protein crystallography, nuclear magnetic resonance (NMR), and XAS can be used to obtain information on metal binding sites in transporters. For example, Leitenmeier *et al.*^82^ isolated and purified TcHMA4 from *Thlaspi caerulescens* (now called *Noccea caerulescens*), added Cd to the protein, and acquired XAS spectra from the Cd that was bound to the protein. Most of the Cd was bound to S of cysteine while binding to histidine (O/N) played a subordinate role. Hence, TcHMA4 would be expected to preferentially bind light Cd isotopes at isotope equilibrium.^43^ To further advance the knowledge on metal isotope fractionation with membrane transport, controlled experiments with distinct membrane proteins^73^ and defined metal transfer rates need to be conducted.

In summary, the XAS-isotope approach has been applied in contaminated or metal-rich environments to investigate the root-to-shoot transport of metals. In these environments, EXAFS gives information on metal sequestration processes in roots. Complementary to EXAFS, isotope fractionation provides an insight on the reactions between the sequestrated metals (e.g. Cu(I)-S, stable Cd–S complexes) and cellular ligands (e.g. Cu(II)-nicotianamine, weak Cd–S complexes), which facilitate the translocation of metals from roots to shoots. Equilibrium isotope fractionation between bound and hydrated metal ions provides a basis for the use of XAS-isotope data in roots, as illustrated by the example of Cd. Here, the use of XAS-isotope data could be effectively complemented with (i) gene expressions for putative ligands and membrane proteins that are involved in translocation and (ii) experimental quantification of isotope fractionation by membrane transporters using plant and/or yeast mutants.

### Translocation within the shoot

A few studies have applied the XAS-isotope approach to study the transport of metals within shoots. Li *et al.*^83^ studied potassium (K) in trees (*Macadamia integrifolia)*. Among the different plant parts, fresh leaves were the heaviest, while dead leaves had the lightest isotope compositions (Table 3). The K concentrations were 3–13 times higher in fresh leaves than in dead leaves. XAS analysis showed that fresh leaves had a higher fraction of K-pectate (60–64%) than dead leaves (42–50%), while the remaining K was present as free K^+^ (i.e. hydrated K^+^). Furthermore, the proportion of K bound to pectate correlated with the enrichment of heavy K isotopes in the different tissues. Pectates provide COO^−^ sorption sites for K that are considered to bind heavy K isotopes. The enrichment of heavy isotopes in young leaves was explained by K remobilization from older to younger leaves. Therefore, it was suggested that K was probably remobilized from old to young leaves as heavy K organic complexes (e.g. mobile K-pectate complexes). Briefly, this study showed that K isotope fractionation within plants may be related to K speciation in different plant parts. However, more studies are needed to investigate, for instance, the role of membrane transporters on K isotope fractionation.

In Ni hyperaccumulating trees (*Rinorea cf. bengalensis*), Zelano *et al.*^32^ found contrasting fractionation patterns as a function of the age of the trees. In one year old trees, leaves accumulated Ni and were strongly enriched in light Ni isotopes compared to roots (Δ^60/58^Ni_leaves–root_ = –0.51 to –0.28‰, Table 3). On the contrary, in 3-year-old trees, leaves were heavier than roots (Δ^60/58^Ni_shoot–root_ = 0.26–0.40‰, Table 3). Ni speciation in leaves was identical in both trees, with Ni bound to low molecular weight complexes with O ligands such as citrate and malate. The authors proposed that in young trees, Ni was rapidly translocated from roots to leaves, whereas in old trees, loading and unloading cycles through phloem-mediated redistribution led to homogenous isotope compositions within the trees. In addition, Ni isotope fractionation through binding to low-molecular weight-organic ligands such as citrate was small (<0.2‰^84^). Based on these first Ni studies, Ni speciation may play a subordinate role for Ni isotope fractionation in Ni hyperaccumulating plants. To further investigate the relation between Ni speciation and Ni isotope fractionation, Ni speciation and isotopes could be measured in additional plant tissues to leaves.

Cereal grains are enriched in heavy Cd isotopes compared to stems and leaves.^54^ To gain more insights on the role of Cd storage forms (i.e. Cd speciation) on isotope fractionation shoots of cereals, speciation and isotopes were measured at flowering and maturity in rice.^33^ Grains were strongly enriched in heavy isotopes compared to the shoots (Δ^114/110^Cd_grain–shoot_ = 0.66‰, Table 3). The speciation of Cd in shoots evolved during maturation (i.e. between flowering and maturity) from mostly Cd–S species (75%) to mostly Cd–O species (80%), whereas in node I and grains, Cd–S represented 100% and 85%, respectively. The enrichment of heavy isotopes in grains was ascribed to a small fraction of Cd that was mobilized from Cd–O pools in the senescing stems and leaves towards the grains via the phloem (Fig. 4). The isotope compositions in node I (the most upper node), flag leaves, panicles, and grains further indicated that heavy Cd isotopes were preferentially transferred from the xylem to the phloem in node I. Nodes act as hubs for the distribution of nutrients and contaminants by connecting xylem and phloem tissues such as stems, leaves, and grains.^75^ The hypothesis that heavy Cd is transported from the xylem to the phloem in nodes was corroborated by Zhong et al. (2021)^85^ who found a relation between the overexpression of the genes *OsHMA2* and *OsLCT1* and the enrichment of heavy isotopes in rice grains. These genes encode membrane transporters that transfer Cd from the xylem to the phloem. The overexpression of *OsHMA2* and *OsLCT1* was detected in node I (the most upper node) as well as in nodes II and III (between roots and node I).^75^ These results suggest that the membrane transporters OsHMA2 and OsLCT1 in the nodes contributed to the enrichment of heavy Cd isotopes in the grains. Moreover, Cd–S binding was conserved when Cd was transported from nodes to grains, as isotope compositions between node I, panicles (connecting nodes and grains), and grains were not distinguishable.^33^ In addition, the speciation in node I and grain was not distinguishable. In contrast, Zhong et al (2021)^85^ found that grains were enriched in heavy isotopes compared to nodes I, II, and II. These conflicting results illustrate that other factors such as distinct Cd and nutrient supply as well as different cultivars may influence the Cd isotope fractionation from nodes to grains.

In conclusion, the combined XAS-isotope approach administered original insights about the complex interplay of sink–source relations (i.e. xylem and phloem translocation), membrane transport, and speciation during the translocation of Cd from stems and leaves into grains and more generally during metal transport in plant shoots.

### Special focus on isotope mass balances and modelling in plant

The metal isotope composition has been modelled using a Rayleigh model in plants^73^ and a box model in unicellular organisms.^22,23^ To this end, isotope fractionation data induced by the complexation of metals were integrated into the parameterization and/or interpretation of these two types of models. The Rayleigh model serves to describe the progressive sequestration of metals in roots along their translocation towards the xylem (Fig. S1). Fitting isotope data with the Rayleigh model yields the isotope fractionation (Δ_storage_) between the sequestered and mobile metal (i.e. the metal species that can be translocated to the shoots). For transition metals, hydrated metals likely represent a minor fraction in the cytosol, and low-molecular-weight ligands are involved in intracellular buffering of metals and facilitation of long-distance transport in xylem and phloem.^81^ Therefore, the Δ_storage_ value can reflect the isotope fractionation between the sequestered metal and the metal complexes involved in metal translocation. Furthermore, Δ_storage_ can include a component that is related to cross-membrane transport (see section ‘Root to shoot translocation’). Data for Zn, Cd, and Ni for root-shoot translocation produced a good fit with the Rayleigh equation (Table S2). Positive Δ_storage_ values for Zn^21,86^ indicated that the sequestration of Zn in roots favoured heavy isotopes. This Δ_storage_ values were similar for both studies (0.37 and 0.32‰) and were ascribed to Zn precipitation and adsorption in the root apoplast as well as compartmentalization in the root vacuole.^21^ In contrast, negative Δ_storage_ values for Cd in cereals^19,46,49^ indicated that sequestration of Cd in roots favoured light isotopes. The Δ_storage_ values were not distinguishable between wheat grown in pots and in the field (–0.22 and –0.26‰) but differed from rice grown in contaminated soils (–0.08‰) (Table S2). Similarly, for the Cd translocation from roots to leaves in cacao seedlings, Moore *et al.*^73^ also reported negative Δ_storage_ values in root and stem (–0.13‰). The Δ_storage_ values of all plants were negative but showed a rather large range. These results suggested that mechanisms controlling the retention of Cd in roots may differ with plant species and metal status in plants.

The Rayleigh model has also been applied to predict the isotope composition within wheat shoots.^19,46^ However, fitting the isotope data with a Rayleigh equation indicates in this case that the partitioning of Cd isotopes in the shoot towards the grain is not simply induced by a Rayleigh-type fractionation (Table S2). This observation is consistent with the complexity of metal translocation pathways and processes in shoots, as summarized in Fig. 4. Furthermore, during the maturation of cereals or the development of fruits in trees, metal concentrations and isotope compositions can significantly alter in shoot organs such as stems and leaves^74^ through changing (i) xylem and phloem fluxes and thereby also changing sink–source relationships among plant organs,^87^ (ii) transporter gene expression,^85^ and (iii) metal speciation.^32,33^ Hence, such dynamic changes in Cd translocation pathways at different growth stages are difficult to integrate into a Rayleigh model. A more detailed description of the Rayleigh model can be found in Section S1 ‘Rayleigh model’.

A box model has been proposed to account for the metal isotope mass balance of fungi or cyanobacteria.^22,23^ The Mg isotope composition of a cyanobacteria cell was modelled as a function of Mg influx (set by the isotope composition of Mg in solution), outflux (set by the isotope composition of free, i.e. uncomplexed Mg in cell), and cell storage (set by isotope composition of different Mg species such as chlorophyll, ATP, and free Mg) (Fig. S2A). Pokharel *et al.*^22,23^ proposed to apply the principle of this model on Mg isotope fractionation in roots. The model implied the following assumptions: (i) an isotope equilibrium between the different metal species in the root, (ii) a homogeneous metal pool of each metal species throughout the root, and (iii) no isotope fractionation during uptake and compartmentalization. For trace metals in roots, these assumptions are not fulfilled (see sections ‘The soil–soil solution–plant interfaces’ and ‘Root to shoot translocation’, Figs. S2B and S3). A more detailed discussion on the box model and its adaptation to roots can be found in Section S2. Box models can provide a basis for modelling the metal isotope translocation in shoots.^23^ However, the model would need adaptation for integration of the several main pools interacting with each other (such as roots, young and old leaves, stems, and seeds). In addition, the changes in metal fluxes within the plant that include dynamic sink–source relationships among the plant organs need to be taken into account. Such dynamic modelling of metal isotope ratios has been proposed in the human body by Jaouen *et al.*^88^

### Metal status in plants impacts isotope composition and speciation

An important factor to consider when applying the combined XAS-isotope approach in plants is the status of the plant in terms of nutrition and toxicity. The micronutrient status of a plant can be deficient, sufficient, supplemented, and toxic, while the status of non-essential metals can be non-toxic and toxic. This status can trigger specific mechanisms that adapt the acquisition, the translocation, the remobilization, and the detoxification of metals in plants, such as the synthesis of phytochelatins to chelate and detoxify metals.^80^ These mechanisms differ between excluders and hyperaccumulating plants.^89–92^ As shown in the previous sections, the detoxification mechanisms can affect the isotope fractionation in plants. For instance, Zn deficiency led to the preferential translocation of heavier isotopes in shoots of rice,^62^ while Zn excess enhanced the accumulation of isotopically light isotopes in aerial parts compared to the control treatments.^93^ The extent of the isotope fractionation between shoots and roots was in some cases affected by the metal supply,^7^ and in other cases not.^65^ In rice, the isotope fractionation between roots and shoots was less affected by the Cd phytoavailability than by the capacity of different rice cultivars to sequestrate Cd in vacuoles.^49^ The distinct isotope fractionation among the different rice cultivars can be explained by a combination of isotope pool size effects and changes in the expression of genes that encode membrane proteins and control the synthesis of ligands at various steps of metal uptake and translocation. Thus, the metal exposure conditions and the nutrient status of plants should always be kept in mind when the speciation and isotope fractionation of different soil–plant systems are compared.

Given that the trace metal concentrations in plants are important to consider when applying the combined XAS-isotope approach, the detection limit of each method is crucial. This is particularly the case for XAS. The detection limit of XAS' depends on the element, edge, and matrix composition. With classical fluorescence detectors, the current limit is around 10 mg kg^−1^ for XANES and around 100 mg kg^−1^ for EXAFS,^94,95^ and it can be reached on a few beamlines worldwide. Consequently, most soils and plants that have been analysed by XAS and the combined XAS-isotope approach originate from contaminated environments (see Sarret et al. (2013)^31^ and this review). The current knowledge is thus ‘biased’ towards plants that cope with high exposure levels of metals. Recent technical advances will extend the scope of the XAS-isotope approach towards less contaminated conditions (see below).

### Methodological advices

For methodological advices specific to isotopes, we refer to the review of Wiggenhauser et al. (2022).^54^ For XAS, detailed recommendations can be found Castillo-Michel et al. (2017).^24^ Here, we want to underline that the data obtained on speciation (XAS) and isotope compositions should be reported in a transparent and consistent way to facilitate the interpretation of the data. For instance, for soil–plant systems, biomass of plant organs studied, and the dry weight concentration of the metal concerned should be reported. The level of concentration should be clearly defined by describing the status of a soil–plant system (e.g. deficient, sufficient, and toxic). Moreover, the number of replicate samples used for each method should be given to assess the biological variation of the system. The biological variation can override the analytical variation; therefore, the biological variation is often more relevant than the analytical variation.^96^

The choice of the compartments of soil–plant systems to be analysed depends on the scientific questions and plant species analysed. For instance, the root apoplast can be analysed indirectly through chemical extractions. These methods involve grinding and centrifugation^97^ and can therefore induce redistributions of metals between the symplast and the apoplast. Salt as extractants^18,47,68^ likely extract weakly bound metals present on the root surface, whereas strong acids may extract the root plaque and a larger part of the apoplastic metal pool.^18,47,69,85^ Such aggressive extractions may damage the cell membranes and thereby cause a release of metals from the symplast into the apoplast. Whatever extractant is used, the extracted pool is an operationally defined pool and needs to be interpreted accordingly (see section ‘The soil–soil solution–plant interfaces’).

For the combined XAS-isotope approach, aliquots of the same sample should be analysed for XAS and isotopes to exploit the complementarity of the techniques. However, the two methods require completely different sample preparation. Generally, XAS analysis on biological tissues should be done in frozen hydrated state. Hence, aliquots should already be taken from fresh samples. In contrast, isotope measurements of the metals presented in this review were prepared with dried samples. An additional difference is that both techniques have quite distinct detection limits. Isotopes can be detected in soil and plant material down to 0.1 mg kg^−1^ DW. In contrast, the detection limit for XAS is higher (see previous section and Future directions and conclusions).

As XAS is the limiting technique in terms of detection limit, optimizing the detection of emitted X-rays is crucial. The detection limit strongly depends on the element and beamline characteristics. This includes the photon flux and the detector efficiency, which are parameters to be verified when selecting a beamline.^24^ Metals can be studied by XAS at the K- or L-edge, and each one has its advantages and disadvantages. As the L-edge is generally more structured than the K-edge, more information can be obtained in the XANES part of the spectra, whereas the EXAFS part is more exploitable at the K-edge. Concerning data acquisition, advantages of K-edge include a higher fluorescence yield and less interference, whereas elastic scattering is generally lower in the low energy range (L-edge) compared to the high energy range (K-edge). For a given shell of an atom, XAS is more sensitive to elements of high atomic number than low atomic number. Also, the matrix of the sample is an important parameter to consider. For frozen, hydrated samples, the high water content can complicate the measurement due to elastic scattering.^98^ Dehydrating the sample by freeze-drying or air-drying can reduce these effects and increase the metal concentration. However, it should be rigorously tested whether the metal speciation changes during the dehydration procedures. Furthermore, the detection limit can be improved by increasing the thickness of the pellet (i.e. the sample powder pressed) that is exposed to the X-rays. However, the optimal thickness of a pellet for fluorescence is restricted by self-absorption effects^14^ and by the geometry of sample holders that typically limits the thickness of the pellet to a few millimetres. Next to consider is the angle at which the pellet is exposed to the X-rays. Classically, spectra are recorded with an incident angle of 45°. In grazing incidence mode, the angle is reduced down to 1°, which can significantly increase the fluorescence yield by increasing the penetration depth of the rays.^99^ Such an increase requires that the diameter of the pellet (or the length of sample exposed to the beam) is increased from typically 5 mm to more than 1 cm. Note that, depending on the sample composition, an increase in elastic scattering may be observed in grazing incident mode and can overrule the gain in fluorescence yield.

We advise to be critical when XAS speciation and isotope compositions are linked to each other, as a relation does not necessarily indicate a causal link. For abiotic systems at equilibrium, the XAS speciation in the solid phase can be directly compared to the equilibrium isotope fractionation between the solid and aqueous species. For biotic systems, isotope fractionation between compartments may be related to changes in speciation in each compartment, but also to pool size effects and to transmembrane transport. Moreover, one critical point is that XAS cannot detect chemical species accounting for less than 10% of the total metal species. XAS provides a snapshot of the major species of metals that accumulated in plant tissues. However, XAS does not probe all metal species that were involved in the transport of metals since they can represent minor and/or transient species. Hence, such minor and/or transient metal species that are potentially important regarding metal transport and isotope fractionation (see previous section) can be overlooked with XAS.

By knowing these limitations of the XAS-isotope approach, future work should better constrain the interactions in the soil and plant compartments studied by testing various environmental conditions (e.g. supply of metals and major nutrients) and analysing different compartments at different timepoints. In plants, the use of complementary genetic techniques like mutant plants and/or the evaluation of gene expression may constrain the system even more and open perspectives to study the role of specific proteins or transporters.

### Future directions and conclusions

The separate approaches of XAS and metal isotope fractionation are well recognized to provide unique information on the fate of metals in the environment.^8,31,54,100^ Here, we reviewed studies that used XAS and isotope ratio analyses combined. The XAS-isotope approach on single phases confirmed the rule of thumb that relates an enrichment of heavier isotopes with a lower coordination number in the case of Zn. However, no relationship between the Zn isotope fractionation and mean bond length for the first coordination shell was found. This suggests that individual bonds, higher shells, and/or other structural parameters play a major role for isotope fractionation. For Ni, XAS provided a structural interpretation for the loss of heavy isotopes during weathering as light isotopes were incorporated into Fe-oxides.^32,44^ In Zn- and Cd-contaminated soils, the formation of Cd–S and Zn–S species in flooded soils retained light isotopes in the solid phase and led to a shift towards heavy isotopes in plants.^49^ Some studies gained novel insights by combining chemical extractions with the XAS-isotope approach for soils and roots (see section ‘The soil–soil solution–plant interfaces’). These extractions helped to distinguish between operationally defined pools and to better decompose isotope fractionation steps along the pathway of metals from the soil to the plant. Moreover, the XAS-isotope approach improved the understanding of processes that control metal uptake and translocation in plants. This included the changes in speciation, redox state, and transmembrane transport and how these processes potentially control the pathway of metals from roots to grains. These highlights indicate the potential of the combined XAS-isotope approach to advance our understanding of biogeochemical mechanisms that control the fate of metals in the environment. However, the combined XAS-isotope approach has been used in a rather exploratory way, i.e. to explore its potential and evaluate how XAS can help to interpret isotope fractionation and vice versa. This section provides a list of research gaps and how they can be filled in order to expand the scope of the XAS-isotope approach.

The knowledge on isotope fractionation in single phases such as minerals, oxides, and soil organic matter is still limited. This lack of knowledge limits the interpretation of the data from more complex systems such as soils and plants. In such systems, multiple processes can determine the changes in concentration, speciation, and isotope fractionation between compartments such as soil pools or plant organs. To disentangle this complexity, several directions can be proposed. More basic knowledge of isotope fractionation by single phases would help to tackle more complex systems. To fill the gap between single phases and soils, some composite systems could be investigated, as recently done Gou et al. (2022)^101^ on a binary system with two types of minerals. Mixtures of mineral and organic phases, or ternary systems that include microorganisms^102^ could be studied as well. A suitable model system to investigate the role of speciation and isotope fractionation could be biofilms that have been used to determine the role of enzymatic versus abiotic reduction of U in the environment.^103^ Finally, the integration of complementary biological, geochemical, and analytical techniques would be required (see below).

A major bottleneck concerns the isotope fractionation during membrane transport in living organisms. To progress on this knowledge, an ideal model system could be unicell organisms.^54^ Applying genetic approaches in unicellular organisms, such as transforming yeast by inserting a specific membrane transporter^73^ or by modifying its gene expression^72^ allows to identify isotope fractionation for cross-membrane transport of specific proteins. Currently, there is a lack of data for efflux transporters such as HMA3 that transport trace metals from the cytosol into the vacuole. First attempts to insert this transporter into yeast to determine its isotope fractionation factor were less conclusive.^73^ For this type of experiment, the validation of the transformation by mapping the subcellular distribution of the studied metal is essential. To quantify actual metal uptake rates (i.e. not net uptake rates) upon the modification of yeast, enriched stable isotopes could be used.^104^ Enriched stable isotopes could also be used in combination with stable isotopes to determine input and output fluxes of metals in plant organs.^105^

Beyond the analysis of metal speciation in bulk samples, micro- and nano-XAS techniques are emerging. These techniques are used in tandem with μXRF and nanoXRF and allow to study metal distribution and speciation in specific soil interfaces and in cellular compartments of plants.^24^ These techniques are now available in almost every synchrotron facility, although generally oversubscribed. A key issue to consider is radiation damage since photons are concentrated in a small spot. Cryogenic conditions are essential to limit these effects, although they may not be sufficient. Therefore, it is essential to evaluate possible changes in metal speciation during sample radiation for each experiment. Laser ablation (LA) coupled with MC-ICP-MS is an emerging technique to determine isotope compositions *in situ* with a lateral resolution of a few micrometers. This combined LA-isotope approach was successfully applied on Si isotopes in plant phytoliths.^106^ Its applicability to soil and plant compartments that are enriched in metals, such as precipitates and root plaque could be tested.

Most of the studies that used a combined XAS-isotope approach focused on the fate of metals in contaminated systems, while no study focused on systems that are relevant for agriculture and food production. So-called biofortification is a pertinent field of research that comprises all agronomic and breeding actions that seek to provide more nutritious crops by increasing their Fe and Zn content.^5,107^ Studying soil–plant systems in which the Zn and Fe status of the plant is deficient or plant organs contain small quantities of Zn and Fe is challenging. While many studies have shown that Zn and Fe isotope compositions can be analysed in systems with very low concentrations of these metals,^108,109^ to obtain a XAS spectra may be challenging, but feasible.^110,111^ Such measurements can be improved by sample preparation and analytical techniques (see section ‘Methodological advices’). In addition, the continuous improvement of classical fluorescence detectors^112^, and the development of new detection modes such as HERFD-XAS, may further lower the detection limit. With HERFD-XAS, it is possible to record high-resolution XANES spectra in samples with concentrations as low as 0.4 mg kg^−^^1^.^94,95,113,114^ So far, this technique is available on a few beamlines worldwide.^112^

The XAS-isotope approach is a novel tool in the toolbox to investigate biogeochemical processes that control metals in soil–plant systems. Several studies showed how this tool can be used to provide novel insights on single phases such as minerals and organic ligands as well as more complex systems such as soils and plants. Combining the XAS-isotope tool with established approaches like chemical extractions and novel technologies such as mapping techniques helps to disentangle individual processes from each other in complex soil–plant systems. Feeding the experimentally obtained data into models has been largely unexplored. The scope of the XAS-isotope tool can be extended from heavily polluted soil–plant systems to systems that are more relevant for food production. Finally, combining XAS and isotope composition analyses at natural abundance to study soil–plant systems is very integrative as it requires knowledge from several scientific disciplines.

## Supplementary Material

mfad016_Supplemental_File

## Data Availability

The data underlying this article will be shared on request to the corresponding authors. No new data were generated or analysed for this mini review.
