# Supplementary material for: Coupling metal stable isotope compositions and X-ray absorption spectroscopy to study metal pathways in soil–plant systems: a mini review
Source: Metallomics. 2023 Mar 9;15(4):mfad016. doi: 10.1093/mtomcs/mfad016 (PMC10084921; doi:10.1093/mtomcs/mfad016)
Supplement: mfad016_Supplemental_File [file mfad016_supplemental_file.pdf]

Supporting information

Coupling metal stable isotope compositions and X-ray absorption spectroscopy to study metal pathways in soil-plant systems: a mini review

Anne Marie Aucour, Géraldine Sarret, Hester Blommaert, Matthias Wiggenhauser

Table SI 1: Comparison of speciation (XAS) and isotope compositions in soils and plants.

System	El.	Reference	treatment/ compartment	major species XANES/EXAFS	Δ (‰)	Relation between speciation and Δ values
Single phase	Zn	[1] (and references therein)	g-Al2O3 (alumina)	Tetrahedral coordination, mean Zn-O dist. 1.98 A	$\Delta^{66}Zn_{sorbed-aqueous}$ 0.47	The sorption as tetrahedral complexes favored heavier Zn isotopes, and this could be explained by the different coordination and bond lengths with aqueous Zn (octahedral, longer bond length). In the case of octahedral Zn complexes, first shell XAS parameters were not sufficient to explain isotope fractionation.
			Zn-Al layered double hydroxide (LDH)	Octahedral coordination, mean Zn-O dist. 2.06 A	$\Delta^{66}Zn_{sorbed-aqueous}$ 0.02	
	Cd	[2]	several Fe(oxyhydr)oxides	Octahedral coordination, distorted, Cd-O dist of 2.28-2.32	$\Delta^{114}Cd_{sorbed-acqueousl}$ -0.51 to -0.55	Fe (oxyhydr)oxides preferentially sorbed light Cd isotopes which may be related to the distorted structure of the Cd-O octahedron as shown by EXAFS.
Soil	Zn	[3, 4]	non-flooded soil	29% in layered double hydroxides / 31% complexed octahedral / 35% complexed tetrahedral	$\Delta^{66}Zn_{CaCl2.extract\ -bulk.soil}$ -0.07	Isotope ratio of the weakly bound Zn in the soil differs, probably through the distinct speciation in the bulk soil. Particularly, the flooded soil contained Zn sulfides that potentially retained light isotopes in the solid soil phase.
			frequently flooded soil	48% phyllosilicates / 45% complexed tetrahedral / 9% Zn-sulfide	$\Delta^{66}Zn_{CaCl2.extract\ -bulk.soil}$ 0.05	
		[3]	original soil	29% in layered double hydroxides (LDH) / 31% complexed octahedral /	$\Delta^{66}Zn_{DTPA\ -bulk.soil}$ 0.11	DTPA removed heavy Zn isotopes that were tetrahedrally sorbed, thereby, the Zn-LDH fraction proportionally increased. The extraction shifted soil Zn towards light isotopes.

				35% complexed tetrahedral		
			soil after DTPA extraction	40% in layered double hydroxides (LDH) / 32% complexed octahedral / 22% complexed tetrahedral	$\Delta^{66}Zn_{DTPA\text{-bulk.soil.after. extract}}$ 0.16	
		[5]	Zn smelter-impacted agricultural topsoils	24% Zn-humate / 28% Zn illite / 30% Zn/Al LDH / 10% Zn-Talc / 8% franklinite	$\Delta^{66}Zn_{soil\text{-smelter slag}}$ -0.33	The heavier Zn isotope composition in the wooded soil is consistent with the higher proportion slag-derived franklinite that is enriched in heavy isotopes. However, the difference in speciation between the two soils is too small to fully explain the lighter isotope composition of the agricultural soil. This might be related to post depositional processes, particularly of soluble Zn species such as sulfates.
			Zn smelter-impacted wooded topsoil	39% Zn-humate / 20% Zn illite / 13% Zn/Al LDH / 11% Zn-Talc / 17% franklinite	$\Delta^{66}Zn_{topsoil\text{-smelter slag}}$ -0.18	
	Cd	[6]	non-flooded soil	13% Cd-S (thiols), 87% Cd-O	$\Delta^{114}Cd_{soil\text{-solution-bulk.soil}}$ 0.23	Upon flooding, the binding of Cd to sulfur increased while the isotope fractionation between the bulk soil and the soil solution did not change. As Cd was rapidly removed from the soil solution with progressing reducing conditions in the soil, it was difficult to capture the changes in $\Delta^{114}Cd_{soil\text{-solution-bulk.soil}}$.
			flooded soil	80% CdS (sulfide), 20% Cd-S (thiols)	$\Delta^{114}Cd_{soil\text{-solution-bulk.soil}}$ 0.21	
soil-plant	Cd	[6]	non-flooded soil	13% Cd-S (thiols), 87% Cd-O	$\Delta^{114}Cd_{plant\text{-bulk.soil}}$ -0.13 to -0.24	Upon flooding, the binding of Cd to sulfur increased while the isotope fractionation during plant uptake shifted towards heavy isotopes. However, intermediate redox effects during soils flooding may have also contributed to the isotope shift.
			flooded soil	80% CdS (sulfide), 20% Cd-S (thiols)	$\Delta^{114}Cd_{plant\text{-bulk.soil}}$ -0.05 to -0.07	
within plant	Cd	[6]	functional tonoplast transporter	100% bound to organic S (thiols) in roots	$\Delta^{114}Cd_{shoot\text{-root}}$ 0.16 to 0.19	Speciation changes could not be detected while the isotope fractionation changes. Either different Cd-S species induced the isotope fractionation (chelating thiols vs. non-chelating thiols), kinetic isotope fractionation through membrane transport occurred, or Cd induced stress mechanisms.
			non-functional tonoplast transporter	100% bound to organic S (thiols) in rosots	$\Delta^{114}Cd_{shoot\text{-root}}$ -0.02 to 0.08	
	K	[7]	fresh leaves	68-72% bound to pectate	$\Delta^{41}K_{old.leaves\text{-young.leaves}}$ -0.85 to -0.60	Enrichment of heavy isotopes in young leaves correlated with the fraction of K that is bound to pectates. However, it is not clear yet how the K speciation and isotope fractionation relate to sink-source relationship within the plant during K retranslation.
			old leaves	42-50% bound to pectate		

	Ni	[8]	leaves of a one year old tree	hydrated Ni 31%, Ni-malate 42%, Ni-citrate 27%	$\Delta^{60}Ni_{leave\text{-}root}$ -0.51 to -0.28	Ni isotope fractionation within the studied trees changed with tree age while Ni speciation in leaves did not change. Changes in Ni isotope ratios may have occurred through phloem redistribution of Ni with increasing tree age.
			leaves of a three year old tree	hydrated Ni 33%, Ni-malate 42%, Ni-citrate 26%	$\Delta^{60}Ni_{leave\text{-}root}$ 0.26 to 0.40	
	Cu	[9]	tomato	Cu(I)-cysteine/glutathione 58-87%, Cu(II)-histidine 14-30% in roots	$\Delta^{65}Cu_{shoot\text{-}root}$ 0.65 to 1.01	In tomato and oat, Cu(I)-thiols were the major species while the Cu isotope fractionation between root and shoot differed between these plants. The distinct isotope fractionation might be related to redox changes of Cu(I)-thiols to Cu(II) complexes such as Cu nicotianamine in tomato while no or complete redox changes may occur in oat.
			oat	Cu(I)-cysteine/glutathione 78-84%, Cu(II)-histidine 14-30% in roots	$\Delta^{65}Cu_{shoot\text{-}root}$ -0.04 to 0.03	
	Zn	[3, 4]	*Phalaris arundinacea*	31% sorbed/complexed as Zn-O tetrahedral, 64% sorbed/complexed as Zn-O octahedral	$\Delta^{66}Zn_{shoot\text{-}root}$ -0.80	Shoots were more strongly enriched in light isotopes in *P. arundinacea* than in *T.latifolia*. This difference could be related to enhanced binding of Zn to S in *T. latifolia* instead of Zn binding to organic acids (octahedral) in *P. arundinacea*.
			Typha latifolia	34% sorbed/complexed as Zn-O tetrahedral, 30% sorbed/complexed as Zn-O octahedral, 20% as ZnS and Zn thiol	$\Delta^{66}Zn_{shoot\text{-}root}$ -0.40	

Modeling of isotope data

1. Rayleigh models

The general form of the Rayleigh equation states that the δ-value ($\delta_{reactant}$) in a diminishing reservoir of the reactant is a function of the initial isotope ratio ($\delta_{reactant,0}$), the remaining fraction of reactant *F,* and the kinetic (or equilibrium) isotope fractionation Δ for the reaction.

$$\delta_{reactant} = \delta_{reactant,0} + \Delta \ln F \qquad (1)$$

It applies when the reactant and product separate progressively from each other (i.e. once the product is formed, it is prevented from interacting with the reactant).
Δ is the isotope fractionation for the reaction, i.e. between the reactant and instantaneous product.

For the closed system of the reactant and cumulated product (i.e. the sum of instantaneous products separated from the reactant pool), the isotope mass balance gives:

$$\delta_{reactant,0} = \delta_{reactant}\ F + \delta_{cumulated\ product}\ (1 - F) \qquad (2a)$$
$$\delta_{cumulated\ product} = (\delta_{reactant,0} - \delta_{reactant}\ F) / (1 - F) \qquad (2b)$$

Hence, the difference in isotope composition between the final (cumulated product) and the remaining reactant is:

$$\delta_{cumulated\ product} - \delta_{reactant} = -\Delta \ln F / (1 - F) \qquad (3)$$

Thus, the fractionation depends on the remaining fraction of reactant *F* (or on the reaction progress). The Rayleigh fractionation model applies when the reactant and product separate progressively from each other. It can thus be used for kinetically controlled systems, but also for any system in which the product, once formed, is removed from the reactant pool. For instance, it has been used to estimate equilibrium isotope fractionation with the precipitation of solids such as metal sulfides (e.g. [10, 11]).

The Rayleigh-type equation has been used to describe the progressive storage of a metal in roots during its radial transfer to xylem and shoot (Table SI 2). In this case, the average δ-value of the absorbed metal by the plant, represented by the average δ-value of the plant, is taken as starting value δ_{in}. The δ-value of the metal exported from root to shoot (the metal left after root sequestration) is expressed as:

$$\delta_{shoot} = \delta_{in} + \Delta_{storage} \ln F_{shoot} \qquad (4a)$$
$$\delta_{shoot} - \delta_{in} = \Delta_{storage} \ln F_{shoot} \qquad (4b)$$

where $\Delta_{storage}$ represents the isotope fractionation with metal sequestration in root, i.e. between sequestrated and mobile (free or weakly bound) metal in the root and F_{shoot} the metal fraction exported to shoot. The δ-value of the metal accumulated in root, δ_{root}, can be expressed using Equation (2b) as:

$$\delta_{root} = (\delta_{in} - \delta_{shoot}\ F_{shoot}) / (1 - F_{shoot}) \qquad (4c)$$

The Rayleigh model has also been used for Cd translocation (i) from root to leaves in cacao seedlings and (ii) from shoot to grain in wheat (Table SI 2). In the latter case, the starting δ-value in the shoot is taken as bulk shoot δ-value and $F_{grain/shoot}$ represents the fraction of the shoot Cd that is translocated from the shoot to the grain.

$$\delta_{grain} - \delta_{shoot} = \Delta_{storage} \ln F_{grain/shoot} \qquad (5)$$

The Rayleigh fitting parameters for translocation of Zn, Ni, Cd in plants are given in Table SI 2. The data for Zn, Cd and Ni for root-shoot translocation produced a good fit with the Rayleigh equation. For Cd translocation from wheat shoot to grain, the intercept for the linear fit between (δ_{grain} - δ_{shoot}) and $\ln F_{grain/shoot}$ significantly differs from 0. This difference indicates that the partitioning of Cd isotopes in the shoot towards the grain is not simply induced by a Rayleigh-type fractionation (Equation (5)). The Rayleigh fitting parameters are further discussed in the main text.

Table SI 2 : Rayleigh fitting parameters for translocation of Zn, Ni, Cd in plants

	Ref	Species	Rayleigh fit	r^2	Substrate
Root to shoot translocation					
$^{66}Zn/^{64}Zn$	[12]	*Arabidopsis halleri, A. petraea*	$\delta_{shoot} - \delta_{in} = 0.37 \ln F_{shoot} - 0.03$	0.98	Hydroponic
$^{66}Zn/^{64}Zn$	[13]	*Thlaspi arvense, Alyssum murale, Noccaea caerulescens*	$\delta_{shoot} - \delta_{in} = 0.32 \ln F_{shoot} - 0.06$	0.909	Hydroponic
$^{60}Ni/^{58}Ni$	[13]	*Thlaspi arvense, Alyssum murale, Noccaea caerulescens*	$\delta_{shoot} - \delta_{in} = -0.24 \ln F_{shoot} - 0.045$	0.805	Hydroponic
$^{114}Cd/^{110}Cd$	[14]	Wheat	$\delta_{shoot} - \delta_{in} = -0.22 \ln F_{shoot} - 0.03$		Soil, pot
$^{114}Cd/^{110}Cd$	[15]	Wheat	$\delta_{shoot} - \delta_{in} = -0.26 \ln F_{shoot} - 0.01$		Soil, field
$^{114}Cd/^{110}Cd$	[6]	Rice	$\delta_{shoot} - \delta_{in} = -0.09 \ln F_{shoot} - 0.03$		Soil, pot
Translocation to grain in the shoot					
$^{114}Cd/^{110}Cd$	[14]	Wheat	$\delta_{grain} - \delta_{shoot} = -0.46 \ln F_{grain/shoot} - 0.37$		Soil, pot
$^{114}Cd/^{110}Cd$	[15]	Wheat	$\delta_{grain} - \delta_{shoot} = -0.40 \ln F_{shoot/grain} - 0.31$		Soil, field
Translocation from root to leaves					
$^{114}Cd/^{110}Cd$	[16]	Cacao seedlings	$\delta_{leaf} - \delta_{plant} = -0.13 \ln F_{shoot}$		Hydroponic

The curves for the Rayleigh models are shown in Fig. SI 1A for shoot to root translocation of Cd in rice [6]. The fit obtained at the flowering stage with a Rayleigh model (Equation 4b) yielded an isotope fractionation $\Delta^{114/112}Cd_{storage}$ of -0.09%. The green curve represents the modeled difference in isotope composition between the Cd exported to shoot and the Cd taken up as a function of *F* (Equation (4b)), the dashed brown curve the modeled difference in isotopic composition between Cd accumulated in root and Cd taken up (Equation (5)). The data obtained for rice mutants without HMA3 and cultivated in wet conditions (non-flooded) diverged from the Rayleigh curves, hence suggesting a change in sequestration and export to the shoot for these samples.

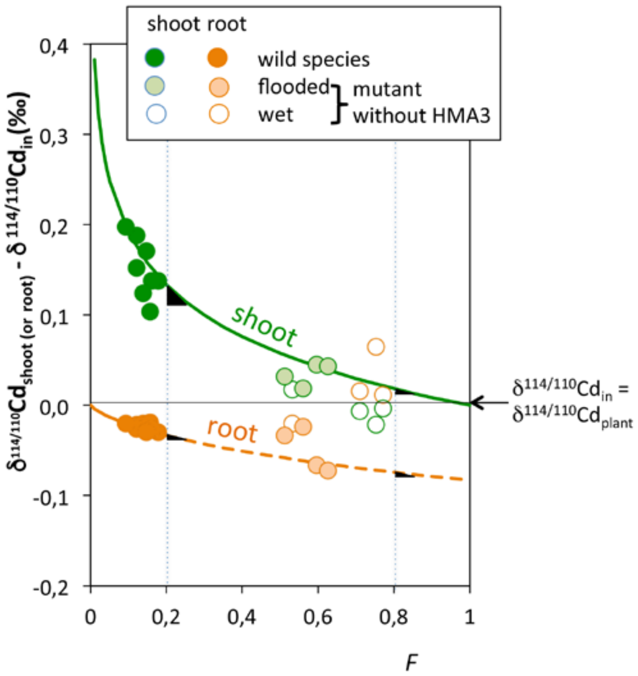

Figure SI 1: Cd isotope data for root and shoot of rice plants [6]. Fitting the data with a Rayleigh model yields an isotope fractionation with root sequestration of -0.09‰. The Rayleigh curves for the isotopic composition of shoot (green curve) and root (orange curve) are shown.

Figure SI 1 illustrates the effect of Cd pool sizes on the isotopic composition. When the translocation to shoot F_{shoot} is limited, the root (the larger Cd pool) is isotopically closer to whole plant than the shoot (the smaller Cd pool) (Fig. SI 1). Similarly, when F_{shoot} is large, the shoot is isotopically closer to the whole plant than the root. Fig. SI 1 also shows how the modeled difference in isotope composition between the shoot and root changes with the translocation to the shoot. It is larger when F_{shoot} is small, i.e. when the fraction of Cd remaining after root sequestration is smaller, which is typical for a Rayleigh process. The modeled shoot and root isotope composition changes for an incremental change in translocation are shown by the black triangles for a small shoot and large root pool (F = 0.2/0.8) and for a large shoot and small root pool (F = 0.8/0.2). Note that in the case of box models (see below), the modeled difference in isotope composition between the shoot and root does not change with translocation if the Cd partitioning in the cell is kept constant. In this latter case, the modeled changes in isotope composition of shoot and root are the same for a given incremental change in translocation.

2. Box models

The isotope composition of metal accumulated in each pool (organ) reflects the isotope mass-balances between the inward and outward metal fluxes.

A simple box model with influx, outflux, cell storage, cell metal speciation has been proposed for Mg isotopes in fungi and Cyanobacteria [17, 18]. The model is based on the isotope mass balances (i) between the different metal species in the cell and (ii) between metal fluxes in and out of the cell. The assumptions are that (i) isotope equilibrium is achieved in the cell between the different metal species, (ii) uptake does not fractionate, i.e. the δ-value of metal taken up by the cell (δ_{in}) equals that of the solution ($\delta_{solution}$) and (iii) that metal outflux (δ_{out}) is isotopically similar to free Cd in the cell (δ_{free}) (Fig. SI 2A).

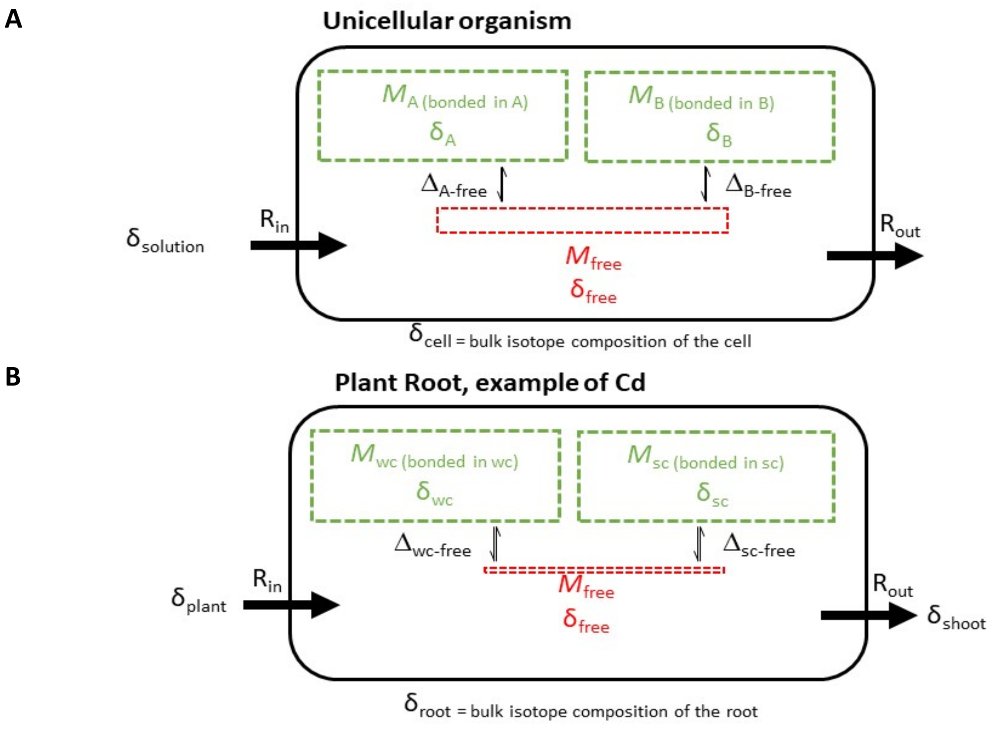

Figure SI 2: Box models for a metal in unicellular organism (A) and for Cd in plant root (B). R_{in} is the flux of a metal into the cell, R_{out} the flux of metal out of the cell. All fluxes (or rates) are given in mol/s. The green pools represent the bonded species, the red pool the free metal (i.e. hydrated metal cation). δ_X is the isotope composition of X, $\Delta_{Z\text{-}Y}$ the equilibrium isotope fractionation between the chemical species Z and Y of the metal. M_Y is the mass of the species Y in the cell. The relative size of each pool is approximately represented. In the case of the Cyanobacteria studied by Pokharel et al. [18], the Mg partitioning in the cell was ca. 5% free Mg, 19% Mg bound to ATP = species A and 76% Mg bound to chlorophyll = species B and the $\Delta_{Z\text{-}Y}$ values between the different species are given in [18].

Following this model, the difference in isotope composition between the cell and the metal in the nutrient solution can be related to the fraction of metal that is bound to organic ligands in the cell f_{bonded}, the equilibrium isotope fractionation between bonded and free metal, and the net flux between cellular metal bonding in the cell and uptake. The model was used to estimate the mass-balance of metals in a cell. This includes an estimation of the flux ratio between storage (i.e. bonded metals) and influx of metals, that derives from measured isotope compositions of (i) the solution, (ii) the cell, (iii) the bonded species and (iv) from the mass fraction of the distinct bonded species.

We aimed to apply the box model that has been designed for a unicellular organism to the root-to-shoot transfer of Cd [6] (Fig SI 2B). To this end, we set the values for $\delta^{114/110}Cd_{root}$, $\delta^{114/110}Cd_{out}$ and $\delta^{114/110}Cd_{in}$ as follows:

- $\delta^{114/110}Cd_{root}$ (measured)
- isotopic composition of Cd efflux from root $\delta^{114/110}Cd_{out}$: $\delta^{114/110}Cd_{shoot}$ (measured)
- isotopic composition of Cd taken up (influx) $\delta^{114/110}Cd_{in}$: $\delta^{114/110}Cd_{plant}$ (calculated from measured δ_{root} and δ_{shoot} values, see Equation (2) of the main manuscript).

For the Cd speciation in root, EXAFS spectra indicate that Cd is fully bound to thiols [6]. Cd^{2+} is known to represent a quite minor fraction of Cd in the cell. Equilibrium isotope fractionation between stable

Cd complexes of Cd with thiols (sc, e.g phytochelatins) or weaker complexes (wc, e.g. glutathione) and free Cd^{2+} (i.e. hydrated Cd^{2+}) was determined using *ab initio* calculations [19].

The modeled difference in isotope composition between root and shoot is related to the equilibrium isotopic fractionation between strong and weak complexes with thiols and free Cd^{2+} as well as on the proportion of strong/weak complexes present (see below for the model equations). Thus, this simple model translates the idea of a direct relation between the metal speciation in root (here, relative proportions of weak and strong complexes of Cd as well as free Cd) and the isotope fractionation that is associated with the translocation from root to shoot (export of free Cd out of the root cell). However, the box model is built-up on several assumptions and simplifications.

1. No isotope fractionation occurs during compartmentalization; isotope fractionation with uptake is taken into account by taking $\delta^{114/110}Cd_{in} = \delta^{114/110}Cd_{plant}$.
2. Isotope fractionation through metal efflux only reflects the isotope fractionation between free and bonded species (i.e. outflux transporter viewed as a funnel for free ion, interaction with transporter binding site neglected); putative outflux through Cd-CAL1 protein secretion [20] is also neglected.
3. There is an isotopic equilibrium between the different metal species in the cell.
4. The sub-pools corresponding to each metal species are considered isotopically homogeneous throughout the root.

Box model estimates for the difference in isotope composition between shoot and root were calculated using $\Delta^{114/110}Cd_{sc\text{-}free}$ and $\Delta^{114/110}Cd_{wc\text{-}free}$ values of -0.8‰ and -0.3‰, respectively [19]. Estimates for the partitioning of Cd between stable and weak complexes with thiols were set at (i) 0.95 and 0.05, respectively or (ii) 0.90 and 0.10, respectively. The box model estimate gives a larger isotopic difference between shoot and root than measured (Fig. SI 3). The observed dampening can be due to isotope fractionation occurring with Cd^{2+} reaction on transporter binding site (either kinetic or related to Cd binding via thiol groups on the transporter) and/or to the transport of Cd-CAL1 protein (Cd-CAL1) towards the xylem and the shoot [20], which were not taken into account in this box model.

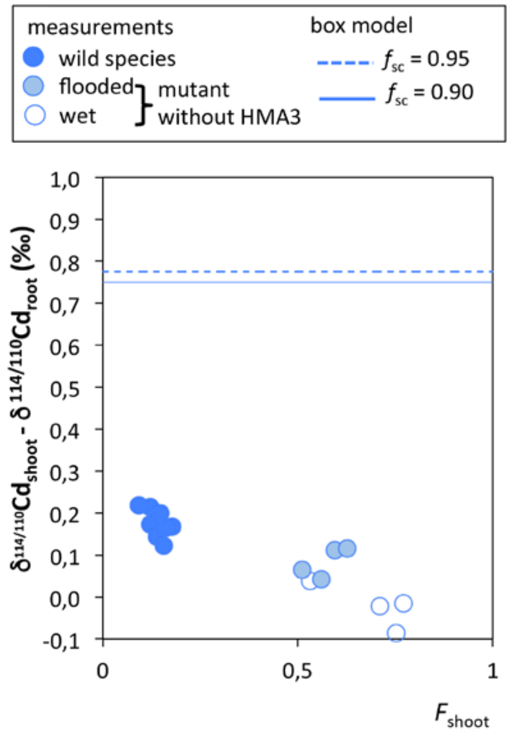

Figure SI 3: Measured values and box model estimates for the difference in Cd isotopic composition between shoot and root of rice. Measured data originate from [6] and are represented by the blue disk

symbols. The box model estimate is shown for Cd partitioning between stable (sc) and weak Cd complexes (wc) with thiols in two cases. f_{sc} and f_{wc} represent the fraction of cellular Cd bonded to stable and weak complexes with thiols, respectively. Two cases are presented. (i) f_{sc}, = 0.90 and f_{wc} = 0.10 (solid blue line) and (ii) f_{sc}, = 0.95 and f_{wc} = 0.05 (dashed blue line). Wild species = rice with functional HMA3 vacuolar transporter, mutant = rice without functional HMA3.

Equations for the model are given below.

A: Box model for unicellular organisms

The isotope mass-balances between free and bonded metal species in the cell is:

$$\delta^{x/y}\mathrm{M}e_{\mathrm{cell}} = \delta^{x/y}\mathrm{M}e_{\mathrm{free}}\,(M_{\mathrm{free}} / M_{\mathrm{cell}}) + \delta^{x/y}\mathrm{M}e_{\mathrm{bonded}}\,(M_{\mathrm{bonded}} / M_{\mathrm{cell}}) \qquad (6a)$$

where $\delta^{x/y}$Me refers to the isotope composition, reported in delta notation (see equation 1 in the main manuscript) of a given metal Me. $M_{\mathrm{bonded}} / M_{\mathrm{cell}}$ represents the mass fraction of Me bonded to e.g. an organic molecule (f_{bonded}), while $M_{\mathrm{free}} / M_{\mathrm{cell}}$ represents the mass fraction of Me that is present as free metal (i.e. hydrated metal ion) (f_{free}).

The metal can be bonded in several different forms, for instance as species A and B. In that case:

$$\delta^{x/y}\mathrm{M}e_{\mathrm{cell}} = \delta^{x/y}\mathrm{M}e_{\mathrm{free}}\,(M_{\mathrm{free}} / M_{\mathrm{cell}}) + \delta^{x/y}\mathrm{M}e_{\mathrm{A}}\,(M_{\mathrm{A}} / M_{\mathrm{cell}}) + \delta^{x/y}\mathrm{M}e_{\mathrm{B}}\,(M_{\mathrm{B}} / M_{\mathrm{cell}}) \qquad (6b)$$

For example, species A and B could represent Mg bound to ATP and chlorophyll or Cd bound to strong and weak sulfur ligands.

The partitioning of the metal between the different chemical species is supposed to be constant in the growing cell.

The rate of change of the metal mass in the growing cell reflects the mass balance between metal influx and outflux.

$$\mathrm{d}M_{\mathrm{cell\ (organ)}} / \mathrm{d}t = R_{\mathrm{in}} - R_{\mathrm{out}} = R_{\mathrm{bonded}} + R_{\mathrm{free}} \qquad (7)$$

where $M_{\mathrm{cell\ (organ)}}$ represents the mass of a metal that accumulated in the cell (in mol). R_{in} is the flux of a metal into the cell, R_{out} the flux of metal out of the cell, R_{bonded} the bonding rate of metal in the cell, R_{free} the rate of free metal formation in the cell. All fluxes (or rates) are given in mol/s.

As the fraction of the free metal is quite small, equation (7) can be rewritten as:

$$R_{\mathrm{in}} - R_{\mathrm{out}} = R_{\mathrm{bonded}} \qquad (8)$$

Similarly to (8), the mass-balance for isotopes writes:

$$\delta^{x/y}\mathrm{M}e_{\mathrm{in}}\,R_{\mathrm{in}} - \delta^{x/y}\mathrm{M}e_{\mathrm{out}}\,R_{\mathrm{out}} = \delta^{x/y}\mathrm{M}e_{\mathrm{bonded}}\,R_{\mathrm{bonded}} \qquad (9)$$

Main assumptions made in [17, 18] are (i) $\delta^{x/y}\mathrm{M}e_{\mathrm{in}}$ equals the $\delta^{x/y}\mathrm{M}e$ value of the metal in the nutrient solution ($\delta^{x/y}\mathrm{M}e_{\mathrm{solution}}$) and, (ii) $\delta^{x/y}\mathrm{M}e_{\mathrm{out}}$ equals the $\delta^{x/y}\mathrm{M}e$ value of the free metal in the cell. Hence the model does not take into account isotope fractionations occuring (i) with uptake and (ii) with the binding of the free metal on the transporters mediating the metal outflux (see Figure 3 of the main text for more details). Based on these assumptions, the mass balance can be written as:

$\delta^{x/y}Me_{solution}\ R_{in} - \delta^{x/y}Me_{free}\ R_{out} = \delta^{x/y}Me_{bonded}\ R_{bonded}$ (10)

Isotope equilibrium is supposed to be achieved between the bonded and free species in the cell, with the difference $\delta^{x/y}Me_{bonded} - \delta^{x/y}Me_{free}$ equal to the equilibrium isotope fractionation $\Delta^{x/y}Me_{bonded\text{-}free}$.

In a further step, the equilibrium isotope fractionation between the bonded and free species, as well Equations (8) and (6a), are integrated in Equation (10). This integration yields:
$\delta^{x/y}Me_{cell} - \delta^{x/y}Me_{solution} = \Delta^{x/y}Me_{bonded\text{-}free}\ (f_{bonded} - R_{bonded}/R_{in})$ (11)

Note, in the case of two bonded species A and B, $\Delta^{x/y}Me_{bonded\text{-}free}$ can be re-written as $\Delta^{x/y}Me_{bonded\text{-}free} = \Delta^{x/y}Me_{A\text{-}free}\ (M_A / M_{bonded}) + \Delta^{x/y}Me_{B\text{-}free}\ (M_B / M_{bonded})$, where M_A / M_{bonded} and M_B / M_{bonded} represent the mass fraction of metals bound to species A and B.

<u>B: Box model for plant root (case of Cd)</u>
As explained above, values for $\delta^{114/110}Cd_{root}$, $\delta^{114/110}Cd_{out}$ and $\delta^{114/110}Cd_{in}$ in the box model were set as follows:

- isotopic composition of Cd stored in root: $\delta^{114/110}Cd_{root}$ (measured)
- isotopic composition of Cd efflux from root $\delta^{114/110}Cd_{out}$: $\delta^{114/110}Cd_{shoot}$ (measured)
- isotopic composition of Cd taken up (influx): $\delta^{114/110}Cd_{plant}$ (calculated from measured δ_{root} and δ_{shoot} values, see Equation (2) of the main manuscript).

Noteworthy, Equation (9) denotes the Cd isotope mass balance in the plant that was used to estimate $\delta^{114/110}Cd_{in}$. Indeed Equation (9) yields:

$\delta^{114/110}Cd_{in}\ R_{in} = \delta^{114/110}Cd_{shoot}\ R_{out} + \delta^{114/110}Cd_{root}\ R_{bonded}$ (12)
R_{bonded}/R_{in} can be roughly estimated from the Cd mass in the root divided by the Cd that has been taken up by the plant, which equals $(1 - F_{shoot})$ (see above in the SI Rayleigh model section for the definition of F_{shoot}), supposing that R_{bonded}/R_{in} remains constant during plant growth. Equation (12) can be rewritten as:

$\delta^{114/110}Cd_{in} = \delta^{114/110}Cd_{shoot}\ F_{shoot} + \delta^{114/110}Cd_{root}\ (1 - F_{shoot})$ (13)

EXAFS speciation results indicate that Cd is fully bound to thiols [6]. Weak and stable complexes with thiol functional groups (R-SH) can thus serve as major Cd pools in the cell (bonded Cd pools). Cd^{2+} represents a quite minor fraction of Cd in the cell. The apoplastic Cd pool and potential structural Cd in the cell wall is neglected.

Theoretical equilibrium isotope fractionation between stable and weak Cd complexes with thiol and free Cd $\Delta^{114/110}Cd_{sc\text{-}free}$ and $\Delta^{114/110}Cd_{wc\text{-}free}$ are given in [19]. The vacuolar transporter HMA3 might also contribute to the isotope fractionation between free Cd^{2+} and exported to shoot and Cd stored in the cell [6], which will be neglected here. The Cd isotope mass-balance in root can thus be written as (with Cd^{2+} being a- minor fraction of Cd in the cell):

$\delta^{114/110}Cd_{root} \approx \delta^{114/110}Cd_{bonded} = \delta^{114/110}Cd_{wc}\ (1 - f_{sc}) + \delta^{114/110}Cd_{sc}\ (f_{sc})$ (14)

where f_{sc} and $1 - f_{sc}$ are the fractions of stable and weak complexes with thiols.

The equilibrium isotope fractionations between the stable and weak complexes with thiols and hydrated Cd^{2+} are integrated in Equation (14), which yields:

$\delta^{114/110}Cd_{root} = \Delta^{114/110}Cd_{wc\text{-}free} + f_{sc}\ (\Delta^{114/110}Cd_{sc\text{-}free} - \Delta^{114/110}Cd_{wc\text{-}free}) + \delta^{114/110}Cd_{free}$ (15)

Supposing that the isotope composition of outflux equals that of free Cd^{2+} in root (as done for cell outflux [18]):

$$\delta^{114/110}Cd_{shoot} = \delta^{114/110}Cd_{free} \quad (16)$$

Then, Equation (15) yields:

$$\delta^{114/110}Cd_{shoot} - \delta^{114/110}Cd_{root} = - \Delta^{114/110}Cd_{wc\text{-}free} + f_{sc} (\Delta^{114/110}Cd_{wc\text{-}free} - \Delta^{114/110}Cd_{sc\text{-}free}) \quad (17)$$

Consequently, the difference in isotope composition between root and shoot is related to the equilibrium isotopic fractionation between stable and weak complexes with thiols and free Cd^{2+} as well as on the proportion of stable to weak complexes present.

References

1. Gou W, Li W, Ji J *et al.* Zinc Isotope Fractionation during Sorption onto Al Oxides: Atomic Level Understanding from EXAFS. *Environmental Science & Technology,* 2018, 52 (16), 9087-96.
2. Yan X, Zhu M, Li W *et al.* Cadmium Isotope Fractionation during Adsorption and Substitution with Iron (Oxyhydr)oxides. *Environmental Science & Technology,* 2021, 55 (17), 11601-11.
3. Aucour A-M, Bedell J-P, Queyron M *et al.* Dynamics of Zn in an urban wetland soil-plant system: Coupling isotopic and EXAFS approaches. *Geochim Cosmochim Acta,* 2015, 160 55-69.
4. Aucour A-M, Bedell J-P, Queyron M *et al.* Zn Speciation and Stable Isotope Fractionation in a Contaminated Urban Wetland Soil–Typha latifolia System. *Environ Sci Technol,* 2017, 51 (15), 8350-8.
5. Juillot F, Marechal C, Morin G *et al.* Contrasting isotopic signatures between anthropogenic and geogenic Zn and evidence for post-depositional fractionation processes in smelter-impacted soils from Northern France. *Geochimica Et Cosmochimica Acta,* 2011, 75 (9), 2295-308.
6. Wiggenhauser M, Aucour A-M, Bureau S *et al.* Cadmium transfer in contaminated soil-rice systems: Insights from solid-state speciation analysis and stable isotope fractionation. *Environmental Pollution,* 2021, 269 115934.
7. Li W, Liu X-M, Hu Y *et al.* Potassium isotopic fractionation in a humid and an arid soil–plant system in Hawaï. *Geoderma,* 2021, 400 115219.
8. Zelano IO, Cloquet C, van der Ent A *et al.* Coupling nickel chemical speciation and isotope ratios to decipher nickel dynamics in the Rinoreacf.bengalensis-soil system in Malaysian Borneo. *Plant and Soil,* 2020, 454 (1-2), 225-43.
9. Ryan BM, Kirby JK, Degryse F *et al.* Copper speciation and isotopic fractionation in plants: uptake and translocation mechanisms. *New Phytologist,* 2013, 199 (2), 367-78.
10. Veeramani H, Eagling J, Jamieson-Hanes JH *et al.* Zinc Isotope Fractionation as an Indicator of Geochemical Attenuation Processes. *Environmental Science & Technology Letters,* 2015, 2 (11), 314-9.
11. Guinoiseau D, Galer SJG, Abouchami W. Effect of cadmium sulphide precipitation on the partitioning of Cd isotopes: Implications for the oceanic Cd cycle. *Earth and Planetary Science Letters,* 2018, 498 300-8.
12. Aucour A, Pichat S, Macnair M *et al.* Fractionation of Stable Zinc Isotopes in the Zinc Hyperaccumulator Arabidopsis halleri and Nonaccumulator Arabidopsis petraea. *Environ Sci Technol,* 2011, 45 (21), 9212-921.
13. Deng T-H-B, Cloquet C, Tang Y-T *et al.* Nickel and Zinc Isotope Fractionation in Hyperaccumulating and Nonaccumulating Plants. *Environmental Science & Technology,* 2014,
14. Wiggenhauser M, Bigalke M, Imseng M *et al.* Cadmium Isotope Fractionation in Soil-Wheat Systems. *Environ Sci Technol,* 2016, 50 (17), 9223-31.
15. Imseng M, Wiggenhauser M, Keller A *et al.* Towards an understanding of the Cd isotope fractionation during transfer from the soil to the cereal grain. *Environmental Pollution,* 2019, 244 834-44.

16. Moore R, Ullah I, de Oliveira V *et al.* Cadmium isotope fractionation reveals genetic variation in Cd uptake and translocation by Theobroma cacao and role of Natural Resistance-Associated Macrophage Protein (NRAMP5) and Heavy Metal ATPases (HMA)-family transporters. *Horticulture Research,* 2020, 7 71.
17. Pokharel R, Gerrits R, Schuessler JA *et al.* Mg Isotope Fractionation during Uptake by a Rock-Inhabiting, Model Microcolonial Fungus Knufia petricola at Acidic and Neutral pH. *Environmental Science & Technology,* 2017, 51 (17), 9691-9.
18. Pokharel R, Gerrits R, Schuessler JA *et al.* Magnesium Stable Isotope Fractionation on a Cellular Level Explored by Cyanobacteria and Black Fungi with Implications for Higher Plants. *Environmental Science & Technology,* 2018, 52 (21), 12216-24.
19. Zhao Y, Li Y, Wiggenhauser M *et al.* Theoretical isotope fractionation of cadmium during complexation with organic ligands. *Chemical Geology,* 2021, 571 120178.
20. Zhong S, Li X, Li F *et al.* Cadmium uptake and transport processes in rice revealed by stable isotope fractionation and Cd-related gene expression. *Science of The Total Environment,* 2022, 806 150633.
